# In Situ Thermal Cross-Linking of 9,9′-Spirobifluorene-Based Hole-Transporting Layer for Perovskite Solar Cells

**DOI:** 10.1021/acsami.3c13950

**Published:** 2023-12-20

**Authors:** Sarune Daskeviciute-Geguziene, Minh Anh Truong, Kasparas Rakstys, Maryte Daskeviciene, Ruito Hashimoto, Richard Murdey, Takumi Yamada, Yoshihiko Kanemitsu, Vygintas Jankauskas, Atsushi Wakamiya, Vytautas Getautis

**Affiliations:** † Department of Organic Chemistry, Kaunas University of Technology, Radvilenu pl. 19, Kaunas 50254, Lithuania; ‡ Institute for Chemical Research, Kyoto University, Gokasho, Uji, Kyoto 611-0011, Japan; § Institute of Chemical Physics, Vilnius University, Sauletekio al. 3, Vilnius 10257, Lithuania

**Keywords:** cross-linking, temperature, hole-transporting layer, perovskite solar cell, spirobifluorene

## Abstract

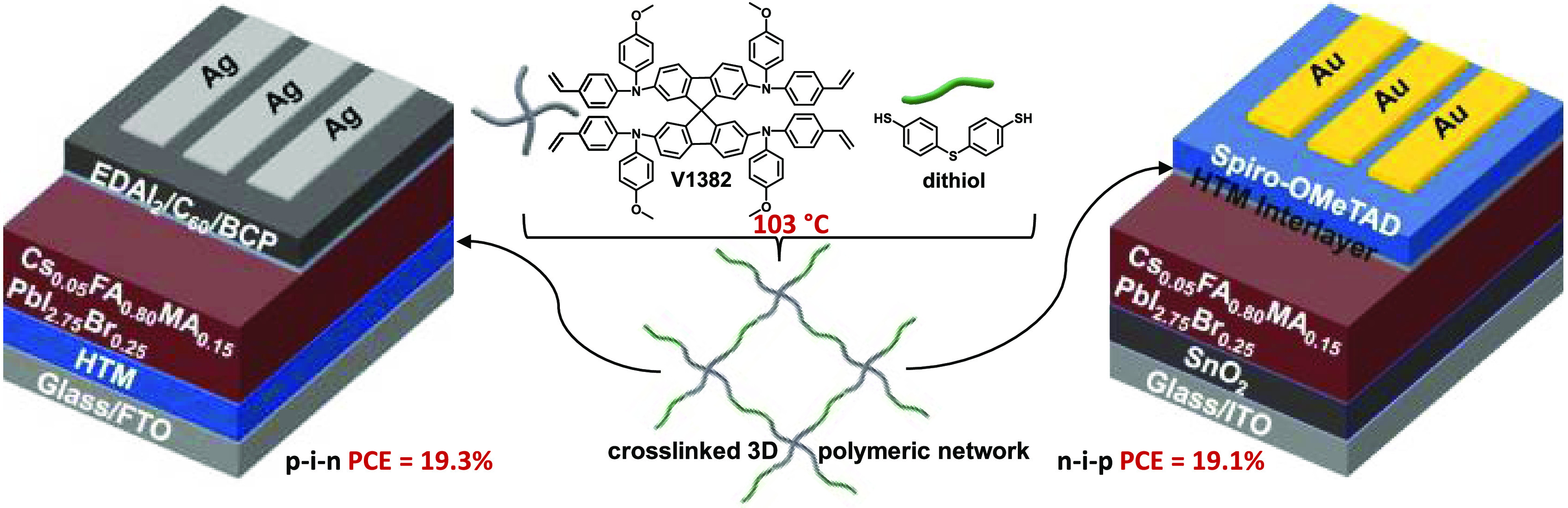

A novel 9,9′-spirobifluorene derivative bearing thermally cross-linkable vinyl groups (**V1382**) was developed as a hole-transporting material for perovskite solar cells (PSCs). After thermal cross-linking, a smooth and solvent-resistant three-dimensional (3D) polymeric network is formed such that orthogonal solvents are no longer needed to process subsequent layers. Copolymerizing **V1382** with 4,4′-thiobisbenzenethiol (dithiol) lowers the cross-linking temperature to 103 °C via the facile thiol–ene “click” reaction. The effectiveness of the cross-linked **V1382**/dithiol was demonstrated both as a hole-transporting material in p–i–n and as an interlayer between the perovskite and the hole-transporting layer in n–i–p PSC devices. Both devices exhibit better power conversion efficiencies and operational stability than devices using conventional **PTAA** or **Spiro-OMeTAD** hole-transporting materials.

## Introduction

Organic–inorganic metal halide perovskite solar cells (PSCs) have received significant interest from the photovoltaic community due to their skyrocketing power conversion efficiencies (PCEs) from 3.8 to 26.1%,^1^ to compete with established solar cell technologies such as crystalline silicon (c-Si) and copper indium gallium selenide (CIGS).^2,3^ Moreover, PSCs may be scaled up using a low-cost solution process from widely available abundant precursors showing promise as a future mainstream photovoltaic (PV) technology.^4,5^ PSCs can also be integrated as top cells into tandem solar cells when combined with existing mature PV technologies to increase efficiency beyond the Shockley–Queisser limit of single-junction devices.^6,7^ However, besides impressive efficiency, the long-term stability of PSC devices under practical working conditions still requires further improvement to satisfy stringent market demands.

A typical PSC consists of a perovskite light absorber sandwiched between an n-type electron-transporting layer (ETL) and a p-type hole-transporting layer (HTL).^8^ Hole-transporting materials (HTMs) play critical roles in efficiently extracting and transporting photogenerated holes from the perovskite layer to the electrode, as well as suppressing charge recombination in PSCs.^9,10^ In general, HTMs should possess the following properties: (1) appropriate energy-level alignment with perovskite materials to guarantee effective hole extraction and electron blocking; (2) high hole mobility; (3) good solubility in common solvents; (4) excellent film-forming ability; (5) good thermal, photochemical, air, and moisture stability; and (6) low cost.^11^ However, the requirements for HTMs vary depending on the device configurations.^12^ For n–i–p PSCs, since the HTM layer is fabricated on top of the perovskite layer, a thick HTM film is required to ensure full coverage of the rough perovskite surface and suppress the diffusion of metal from the top electrode into the perovskite. Also, the HTM film should ideally be hydrophobic to protect the perovskite from moisture ingress. Although various kinds of HTMs have been developed, 2,2′,7,7′-tetrakis(*N*,*N*-di-*p*-methoxyphenylamino)-9,9′-spirobifluorene (**Spiro-OMeTAD**) has been proven to be the most reliable and effective HTM for use in n–i–p PSCs.^13,14^**Spiro-OMeTAD** has a large bandgap (about 3.0 eV) and a relatively shallow highest occupied molecular orbital (HOMO) energy level of around −5.1 eV,^12^ which provides good electronic alignment with perovskite materials. In addition, the synthesis and solution-based film processing of **Spiro-OMeTAD** are well established and are well suited to the fabrication of large-area solar cells. On the other hand, chemical dopants or additives, such as lithium bis(trifluoromethanesulfonyl)imide (LiTFSI), cobalt(III) complexes, and 4-*tert*-butylpyridine (*t*BP), are needed to improve the conductivity and hole mobility of the pristine **Spiro-OMeTAD**. These hygroscopic dopants have an impact on the device’s long-term stability due to moisture ingress and ion migration. Therefore, an interlayer that is hydrophobic^15^ and/or able to block ion migration^16^ between the perovskite layer and HTM layer would be helpful to improve the stability of PSCs.

In the case of p–i–n devices, solution-processing of the perovskite absorber layer puts additional constraints on the choice of HTMs, as the materials must now be made resistant to the perovskite precursor solution, commonly a mixture of polar dimethylformamide (DMF) and dimethyl sulfoxide (DMSO) solutions. So far, polymeric HTMs, such as poly(3,4-ethylenedioxythiophene):polystyrenesulfonate (**PEDOT:PSS**),^17^ poly[3-(4-carboxylatebutyl)thiophene] (**P3CT**) derivatives,^18,19^ and poly[bis(4-phenyl)(2,4,6-trimethylphenyl)amine] (**PTAA**)^20,21^ or combinations thereof, are widely used for this application.^22,23^ Among them, **PTAA**, with its excellent electrical properties and chemical neutrality, has attracted particular interest.^5,24−27^ However, the strongly hydrophobic **PTAA** film surface results in the dewetting of the perovskite precursor solution and low-quality perovskite films.^28^ Despite several attempts to modify **PTAA**, such as chemical doping,^29,30^ surface post-treatment,^31^ and interfacial functionalization,^32^ the tedious synthetic process and batch-to-batch variation of **PTAA** remain significant issues restricting its application to large-scale device fabrication. In this regard, small molecular organic molecules offer potential advantages, such as a well-defined molecular weight, ease of synthesis, and good reproducibility. To insolubilize small molecular molecules, the use of molecules with anchoring groups such as phosphonic acid (−PO(OH)_2_) or carboxylic acid (−COOH) that can spontaneously bind to the transparent conducting oxide surface to form a conformal hole-collecting monolayer has been demonstrated as an effective way by our groups and others.^33−36^ An alternative approach is to polymerize the small molecules in situ via cross-linking reactions. Soluble small molecules bearing cross-linkable units, such as vinyl, acrylate, azide, and oxetane groups, can form insoluble cross-linked three-dimensional (3D) networks under thermal or ultraviolet (UV) treatment.^37−39^ Such cross-linked 3D networks could enable solvent-resistant hole-transporting layers^40−46^ and protective interlayers.^47,48^ However, the reported cross-linkable systems would not be suitable for flexible p–i–n PSCs with film substrates or for n–i–p PSCs due to their high cross-linking temperatures (usually >180 °C), which exceed the tolerance of the underlying layers.

**Scheme 1:**
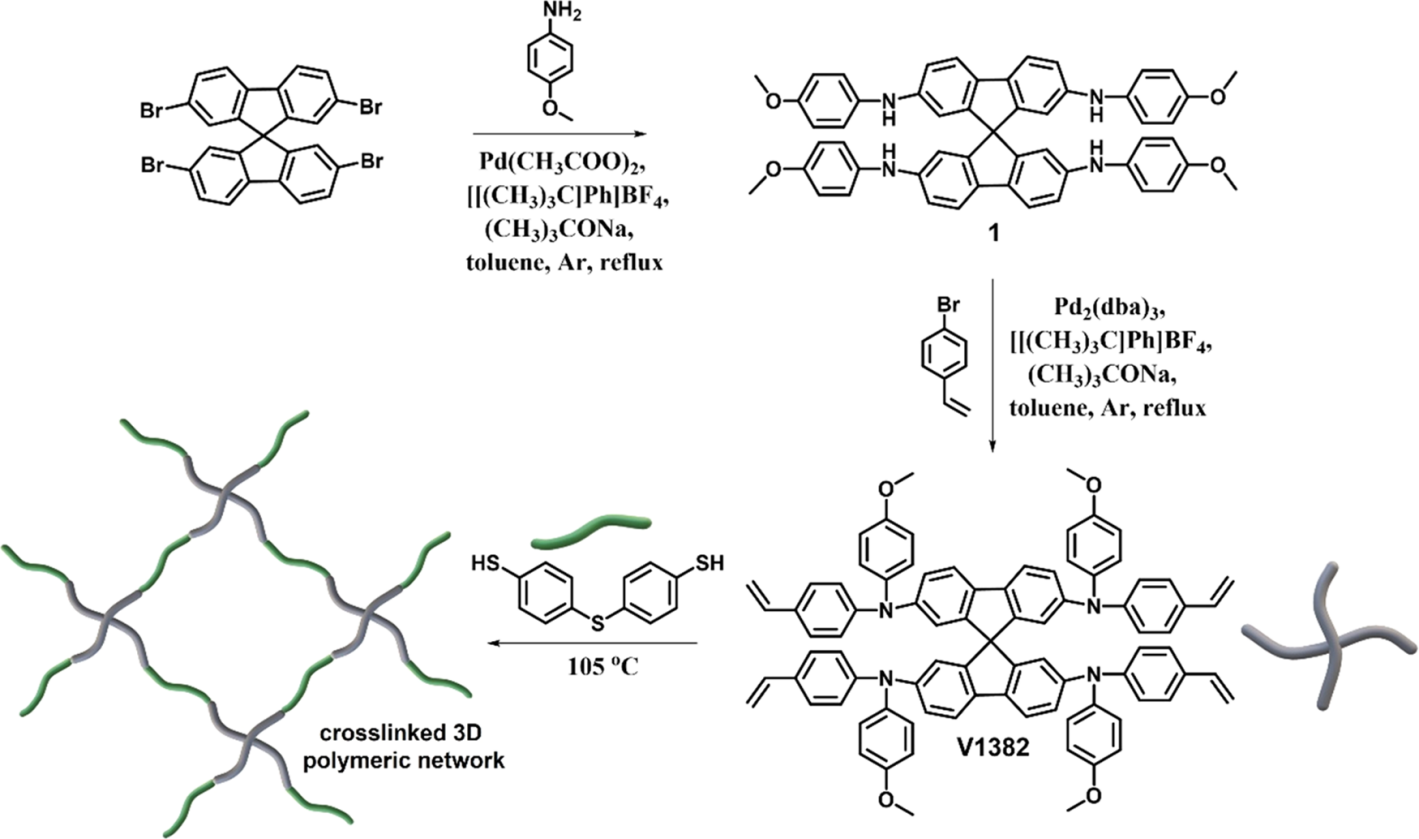
Synthetic Route of the 9,9′-Spirobifluorene Polymer Precursor **V1382** and Its Schematic Thiol–Ene Cross-Linking Using 4,4′-Thiobisbenzenethiol as a Cross-Linker

In this work, we report the development of a 9,9′-spirobifluorene-based molecule functionalized with four vinyl groups (**V1382**) for the targeted cross-linkable HTL (Scheme 1) and its application to PSCs. To lower the cross-linking temperature, the introduction of an aliphatic cross-linker containing four thiol groups, pentaerythritol tetrakis(3-mercaptopropionate) (PETMP), has been reported.^49^ We chose a dithiol-terminated diphenylsulfide, 4,4′-thiobisbenzenethiol, as a cross-linker since it has a shorter insulating part than PETMP and may generate a stable radical form to facilitate the thiol–ene “click” reaction with **V1382**. We found that the cross-linking between **V1382** and 4,4′-thiobisbenzenethiol (dithiol) can occur at a low temperature of 103 °C to form an insoluble 3D polymer network. To the best of our knowledge, this is the lowest cross-linking temperature reported for HTLs for PSCs. Benefiting from the mild cross-linking conditions, this cross-linkable system is suitable for applications in both p–i–n and n–i–p PSC architectures. Devices employing the cross-linked **V1382**/dithiol as the hole-transporting layer in p–i–n PSCs and as the interlayer between the perovskite layer and **Spiro-OMeTAD** in n–i–p PSCs have shown improved performance and long-term stability compared with devices using conventional HTMs. These results demonstrate cross-linking as an efficient strategy for low-cost and high-performance organic semiconducting materials, not only for photovoltaics but also for other optoelectronic devices such as light-emitting diodes, phototransistors, photocells, and so on.

## Results and Discussion

The polymer precursor **V1382**, which possesses a 9,9′-spirobifluorene core and four vinyl cross-linkable groups, was synthesized in a facile 2-step synthetic procedure with commercially available starting materials as shown in Scheme 1. During the first step, the palladium-catalyzed Buchwald–Hartwig amination reaction of 2,2′,7,7′-tetrabromo-9,9′-spirobifluorene and *p*-anisidine was carried out to give aminated precursor **1** in 70% yield. Compound **1** was then vinyl-functionalized by using 4-bromostyrene to generate the target product **V1382** in 51% yield. Structures of the synthesized compounds were characterized by nuclear magnetic resonance (NMR), elemental analysis (EA), and mass spectrometry (MS). The total cost for **V1382** is estimated to be 42 € g^–1^, much cheaper than widely used HTMs,^50^ indicating its strong potential for large-scale manufacturing processes (Table S1). Detailed synthesis procedures and analysis data are given in the Supporting Information.

**Figure 1:**
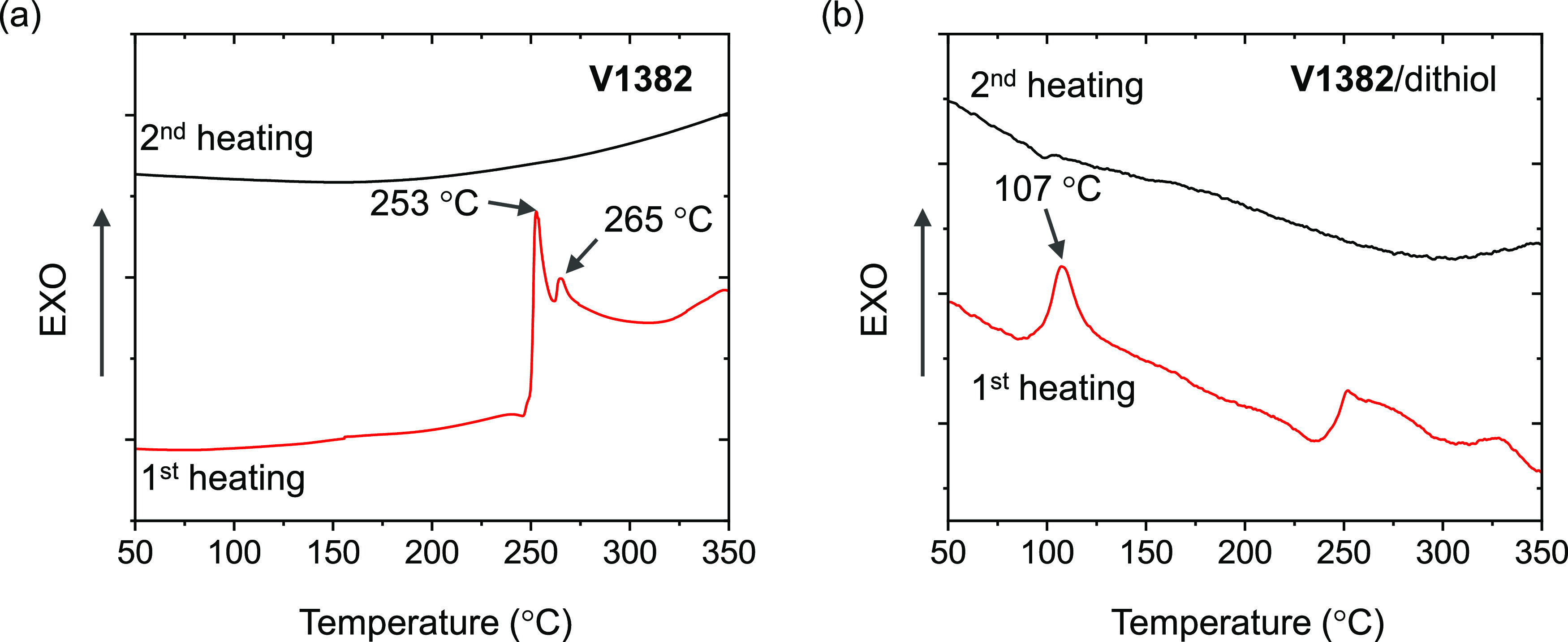
Differential scanning calorimetry curves (scan rate, 10 °C min^–1^; N_2_ atmosphere) of (a) **V1382** and (b) a mixture of **V1382** with 4,4′-thiobisbenzenethiol.

The thermal properties of **V1382** and its cross-linking reaction with 4,4′-thiobisbenzenethiol were investigated by thermogravimetric analysis (TGA) and differential scanning calorimetry (DSC). The decomposition temperature corresponding to 5% weight loss (*T*_dec_) of **V1382** was estimated from the TGA curve to be 460 °C (Figure S1), confirming that **V1382** has good thermal stability. As shown in Figure 1a, an exothermic peak was detected at 253 °C during the first scan, while no distinct phase transition could be observed until 350 °C in the second heating scan, suggesting that thermal cross-linking of **V1382** occurs at 253 °C. In contrast, after mixing **V1382** with a dithiol cross-linker, 4,4′-thiobisbenzenethiol, in a molar ratio of 1:2, the exothermic peak shifted to the region of 103–120 °C, and the cross-linking temperature (*T*_poly_) was detected at 107 °C (Figure 1b). The results imply that the fast thermal cross-linking occurs due to the facile thiol–ene “click” reaction. It is worth noting that this is the lowest cross-linking temperature reported in the PSC field,^40−52^ enabling the application in both p–i–n and n–i–p PSC architectures.

To evaluate the optical properties of **V1382** and formed polymers, ultraviolet–visible (UV–vis) absorption and photoluminescence (PL) spectra were measured from tetrahydrofuran (THF) solutions and thin films. The results are shown in Figure S2 and summarized in Table 1. The absorption maxima (λ_abs_) of **V1382** were observed at 336 and 395 nm. The less intense absorption peak at 336 nm can be assigned to the π–π* transition, while the more intense absorption peak at 395 nm corresponds to the *n*–π* transition. After polymerization of **V1382** at 255 °C, wide absorption band ranging from 275 to 450 nm was observed, while after thermal cross-linking using dithiol at 103 °C, the absorption spectra of the polymer had two main peaks at around 303 and 383 nm. In addition, the emission maxima (λ_em_) of **V1382** were observed at 419 and 441 nm with a Stokes shift value of 24 nm.

**Figure 2:**
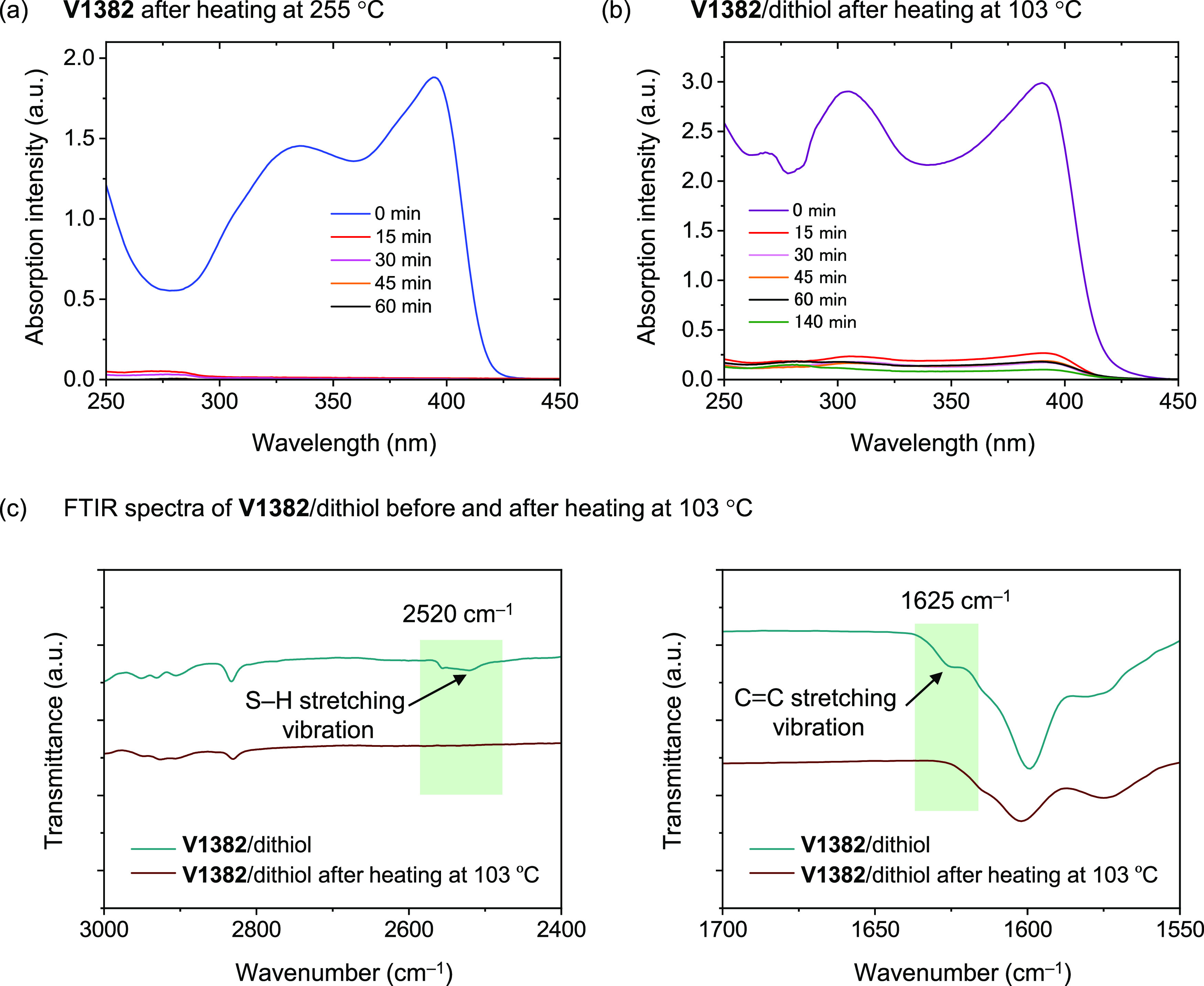
UV–vis spectra of (a) cross-linked **V1382** and (b) **V1382**/dithiol films with different annealing times. (c) FTIR spectra before and after cross-linking of **V1382** with dithiol.

**V1382** films with and without 4,4′-thiobisbenzenethiol cross-linker (molar ratio = 1:2) were prepared by spin-coating the corresponding materials in THF solutions (**V1382** 20 mg mL^–1^). The ability to form insoluble cross-linked networks was evaluated by measuring the UV–vis absorption of these spin-coated films. The results are shown in Figure 2a,b. After annealing the films of **V1382** without and with the dithiol cross-linker for only 15 min at 255 and 103 °C, respectively, and rinsing with THF several times to remove soluble parts, absorbance from the films was still detected. It indicates that the cross-linking of these films occurred under these conditions, resulting in good solvent-resistant films. We note that such rapid cross-linking is quite unusual for thiol–ene-type polymerization according to our previously reported works,^42,44^ suggesting that spiro configuration might be sterically or energetically favorable for this type of reaction. A longer time frame was used to quantitatively cross-link the films. The cross-linking process in both cases was completed after annealing for 60 min.

Fourier transform infrared (FTIR) spectra (Figure 2c) were recorded to ascertain the occurrence of the cross-linking. After **V1382** cross-linking with dithiol at 103 °C, the peak of S–H stretching vibration at 2520 cm^–1^ and the peak of C=C stretching vibration at 1625 cm^–1^ disappeared compared with the peaks before heating confirming that fast thermal cross-linking occurs after heating **V1382** with a dithiol cross-linker at 103 °C.

**Figure 3:**
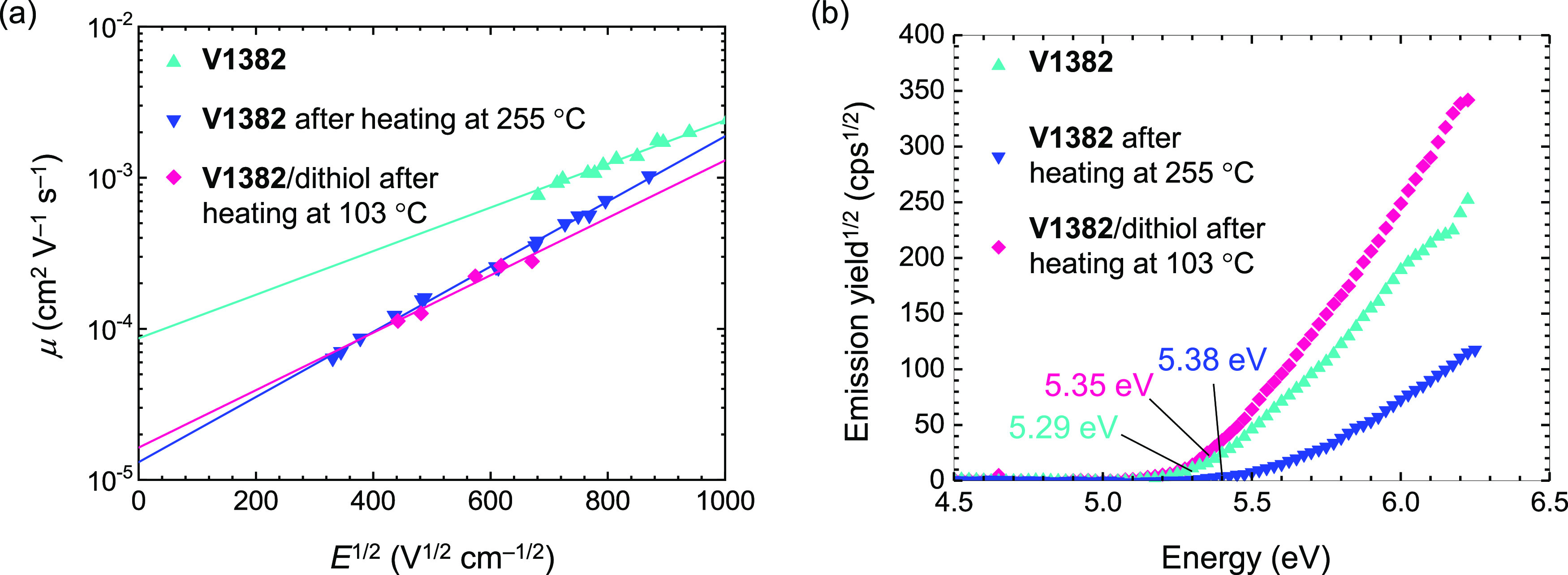
(a) Electric field dependencies of the hole-drift mobilities in charge transport layers and (b) photoelectron yield of **V1382** and cross-linked films measured in air.

The hole-transport properties of the HTMs were characterized with the aid of xenographic time-of-flight (XTOF) measurements (Figure 3a). At zero field strength, **V1382** demonstrates a hole-drift mobility of 8.7 × 10^–5^ cm^2^ V^–1^ s^–1^. After thermal annealing, regardless of using the dithiol cross-linker, the hole mobilities of cross-linked films slightly reduce to 1.3 × 10^–5^ cm^2^ V^–1^ s^–1^, yet are still comparable to those of popular HTMs for PSCs.^53,54^ In addition, the solid-state ionization potential (*I*_p_) of **V1382** and the cross-linked films were measured by using photoelectron spectroscopy in air (PESA). As shown in Figure 3b, the ionization potential of the **V1382** film was measured to be 5.29 eV. The *I*_p_ values slightly increase to 5.38 and 5.35 eV in the case of cross-linked **V1382** without and with 4,4′-thiobisbenzenethiol, respectively. The *I*_p_ values of the cross-linked **V1382** films are smaller than the valence band (VB) of typical perovskite materials such as CH_3_NH_3_PbI_3_ (MAPbI_3_, VB = 5.45 eV) or Cs_0.05_FA_0.80_MA_0.15_PbI_2.75_Br_0.25_ (FA: formamidinium, VB = 5.56 eV)^34^ and larger than those of conventional HTMs such as **PTAA** or **Spiro-OMeTAD**. As shown in energy-level diagrams of both p–i–n and n–i–p PSC devices (Figure S3), compared to conventional HTMs, the smaller energy-level offset between the cross-linked **V1382** and the perovskite suggests that more efficient hole transfer could be expected for the cross-linked **V1382**.

**Table 1:** Thermal, Optical, and Photophysical Properties of **V1382** and **V1382**/Dithiol

	*T*_poly_ (°C)^a^	*T*_dec_ (°C)^a^	λ_abs_ (nm)^b^	λ_em_ (nm)^b^	*I*_P_ (eV)^c^	μ_0_ (cm^2^ V^–1^ s^–1^)^d^
**V1382**	253, 265	460	336, 395	419, 441	5.29	8.7 × 10^–5^
**V1382** after heating at 255 °C^e^	–	–	371	–	5.38	1.3 × 10^–5^
**V1382**/dithiol after heating at 103 °C^e^	–	–	303, 383	–	5.35	1.3 × 10^–5^

a Polymerization (*T*_poly_) and decomposition (*T*_dec_) temperatures observed from DSC and TGA, respectively (scan rate = 10 °C min^–1^, N_2_ atmosphere).

b Absorption and emission spectra were measured in THF solutions (10^–4^ M) or thin films.

c Ionization energies of the films measured using PESA.

d Mobility value at zero field strength.

e After annealing, films were rinsed with THF several times.

X-ray photoelectron spectroscopy (XPS) measurements were carried out to prove the interaction between the cross-linked **V1382**/dithiol and the perovskite (Cs_0.05_FA_0.80_MA_0.15_PbI_2.75_Br_0.25_). Figure S4 presents the XPS spectra of Pb 4*f* peaks in the pristine perovskite film and the perovskite film with cross-linked polymer surface modification. Compared to the pristine film, the peaks of Pb 4f_7/2_ and Pb 4f_5/2_ in the modified perovskite film shifted 0.3 eV to a higher binding energy, implying an interaction between the cross-linked **V1382**/dithiol and the perovskite surface. This could benefit solar cell operational stability.

**Figure 4:**
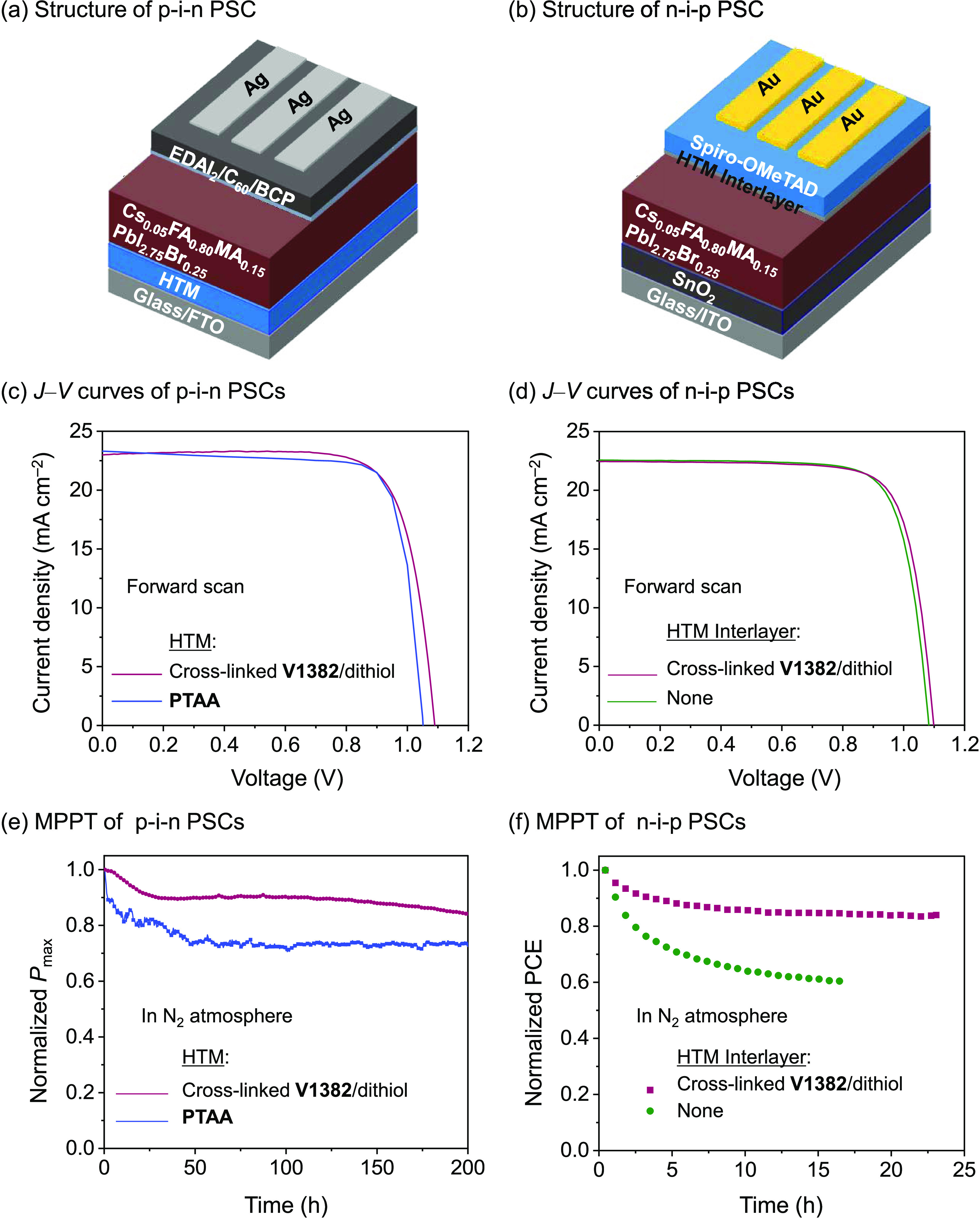
Structure of (a) p–i–n and (b) n–i–p PSCs. (c) *J–V* curves and (e) MPPT of p–i–n PSCs. (d) *J–V* curves and (f) MPPT of n–i–p PSCs.

To evaluate the efficacy of the HTM formed by the cross-linking between **V1382** and 4,4′-thiobisbenzenethiol (named cross-linked **V1382**/dithiol) in PSCs, both p–i–n devices [fluorine-doped tin oxide (FTO)/HTM/perovskite/ethylenediammonium diiodide (EDAI_2_)/C_60_/bathocuproine (BCP)/Ag] (Figure 4a) and n–i–p devices [indium tin oxide (ITO)/SnO_2_/perovskite/(with or without HTM interlayer)/**Spiro-OMeTAD**/Au] (Figure 4b) were fabricated. In the p–i–n PSCs, EDAI_2_ was used as a post-treatment for the perovskite surface to improve the cell voltages.^55^ A triple cation perovskite (Cs_0.05_FA_0.80_MA_0.15_PbI_2.75_Br_0.25_) with a bandgap of 1.56 eV was selected as the light absorber material.^34,56^ Cross-linked **V1382**/dithiol was used as the HTM and the HTM interlayer in p–i–n and n–i–p PSCs, respectively. The details for the device fabrication are provided in the Supporting Information. The current–voltage (*J–V*) curves of devices were measured under AM 1.5G illumination at 100 mW cm^–2^, and detailed device parameters are listed in Table 2.

**Table 2:** Photovoltaic Parameters of p–i–n and n–i–p PSCs Derived from *J–V* Measurements

p–i–n PSC Devices						
HTM^a^	scan^b^	*J*_sc_ (mA cm^–2^)^c^	*V*_oc_ (V)^c^	FF^c^	PCE (%)^c^	HI^d^
cross-linked **V1382**/dithiol	F	23.0 (22.6 ± 0.3)	1.09 (1.08 ± 0.01)	0.77 (0.77 ± 0.02)	19.3 (18.7 ± 0.4)	–0.027
	R	23.5 (22.8 ± 0.4)	1.07 (1.07 ± 0.01)	0.75 (0.74 ± 0.02)	18.8 (18.1 ± 0.8)	
**PTAA**	F	23.3 (21.5 ± 0.8)	1.05 (1.05 ± 0.01)	0.79 (0.77 ± 0.02)	19.3 (17.5 ± 1.0)	–0.090
	R	22.3 (21.1 ± 0.7)	1.06 (1.05 ± 0.01)	0.75 (0.75 ± 0.03)	17.7 (16.6 ± 1.1)	

n–i–p PSC Devices						
HTM^a^	scan^b^	*J*_sc_ (mA cm^–2^)^c^	*V*_oc_ (V)^c^	FF^c^	PCE (%)^c^	HI^d^
cross-linked **V1382**/dithiol/**Spiro-MeTAD**	F	22.4 (22.1 ± 0.4)	1.10 (1.05 ± 0.03)	0.77 (0.75 ± 0.01)	19.1 (17.6 ± 0.8)	–0.032
	R	22.4 (22.1 ± 0.4)	1.09 (1.06 ± 0.02)	0.76 (0.76 ± 0.01)	18.5 (17.8 ± 0.5)	
**Spiro-OMeTAD**	F	22.6 (22.2 ± 0.4)	1.08 (1.05 ± 0.02)	0.77 (0.75 ± 0.01)	18.9 (17.5 ± 0.8)	–0.050
	R	22.5 (22.1 ± 0.4)	1.07 (1.06 ± 0.01)	0.75 (0.74 ± 0.01)	18.0 (17.4 ± 0.5)	

a HTM (**V1382**/dithiol/1:2 molar ratio) were spin-coated on FTO substrates or on top of the perovskite layer from PhCl solution to fabricate p–i–n or n–i–p PSCs, respectively. The optimized concentration of **V1382** is 2.0 and 1.0 mg mL^–1^ for p–i–n and n–i–p PSCs, respectively.

b Forward and reverse indicate the scan direction from *J*_SC_ to *V*_OC_ and from *V*_OC_ to *J*_SC_, respectively.

c The average and standard deviation values were given in parentheses.

d Hysteresis index (HI) = (PCE_Reverse_ – PCE_Forward_)/PCE_Reverse_.

In the p–i–n PSCs, all HTMs are used without any dopants or additives. Devices with **PTAA** as the HTM were also fabricated as references. The performance of the cross-linked **V1382**/dithiol-based devices with different concentrations of **V1382** (0.125–4.0 mg mL^–1^) is presented in Table S2 and Figures S5–S9 in the Supporting Information. The morphology of the perovskite films was characterized with the help of scanning electron microscopy (SEM) (Figures S10 and S11). All of the perovskite layers are smooth and pinhole-free, indicating that the perovskite films are not significantly affected by the concentration of **V1382** used for cross-linking.

The concentration of **V1382** used for cross-linking with dithiol was optimized to be 2.0 mg mL^–1^. Devices with cross-linked **V1382**/dithiol fabricated by using <2.0 mg mL^–1^ of **V1382** exhibited a lower open-circuit voltage and a larger hysteresis, while those using >2.0 mg mL^–1^ of **V1382** showed a lower fill factor. Under the optimized conditions, in the forward scan, the cross-linked **V1382**/dithiol-based p–i–n devices exhibited a short-circuit current density (*J*_SC_) of 23.0 mA cm^–2^, an open-circuit voltage (*V*_OC_) of 1.09 V, and a fill factor (FF) of 0.77, resulting in a PCE of 19.3%. The *J*_SC_ values derived from the *J–V* measurements were consistent with the values integrated from the incident photon-to-current efficiency (IPCE) spectra (Figures S6–S8). Compared to the reference devices based on **PTAA** (Figures 4c, S12, and S13), the cross-linked **V1382**/dithiol-based devices showed comparable PCE (19.3 vs 19.3%), higher *V*_OC_ (1.09 vs 1.05 V), and smaller hysteresis (−0.027 vs −0.090). The higher *V*_OC_ of the cross-linked **V1382**/dithiol-based devices could be attributed to the larger ionization potential (or deeper HOMO energy level), resulting in a better energy alignment with the VB of the perovskite material.

To compare the operational stability of p–i–n devices using cross-linked **V1382**/dithiol and reference **PTAA** HTMs, maximum power point tracking (MPPT) was carried out under AM 1.5G in an inert atmosphere (Figure 4e). The PCE of the **PTAA**-based reference device degraded to 80% of its initial value after 30 h. In contrast, the cross-linked **V1382**/dithiol-based device still retained 84% of the initial output after 200 h, indicating the superior long-term stability of the cross-linked HTMs. In addition, Figure S14 shows the much improved thermal stability of the unencapsulated device using the cross-linked **V1382**/dithiol, which remained at 91% of its initial PCE after being heated at 85 °C in air for 50 h under a relative humidity of 40%, while the PCE of the **PTAA**-based device dropped to 76% of its initial value.

The electrical properties of the devices were investigated with the aid of impedance spectroscopy (AM 1.5G, zero applied bias; Figure S15). The data are analyzed with a simple equivalent circuit comprising series and parallel resistances together with a parallel capacitance element. At low bias voltages, the parallel resistance is determined by recombination and/or leakage currents, with larger values indicating either better quality of the perovskite layer or more efficient charge extraction from the perovskite absorber. The parallel resistance of the cross-linked **V1382**/dithiol-based device was estimated to be 228 cm^2^, higher than that of the **PTAA**-based device (152 Ω cm^2^). It indicates that the interfacial recombination could be suppressed in the case of the device with cross-linked **V1382**/dithiol. The results are in good agreement with the trend in *V*_OC_.

The effect of the cross-linked **V1382**/dithiol as the interlayer between the perovskite layer and **Spiro-OMeTAD** on the performance of the n–i–p PSCs was investigated. In this case, **Spiro-OMeTAD** was doped with LiTFSI, the Co(III) complex, and *t*BP. Devices using the doped **Spiro-OMeTAD** without the interlayer were also fabricated as reference n–i–p devices (Figure S16). The concentration of **V1382** on the cross-linking precursor used for the interlayer was optimized and determined to be 1.0 mg mL^–1^ (Table S3, and Figures S17–S20). As shown in Figure 4d, after optimization, the device with the cross-linked interlayer exhibited a PCE of 19.1% with a *J*_SC_ of 22.4 mA cm^–2^, a *V*_OC_ of 1.10 V, and an FF of 0.77 in the forward scan. For the reference device without the interlayer, slight drops in *V*_OC_ and PCE were observed (*V*_OC_ = 1.08 V and PCE = 18.9%). It implies that by inserting the cross-linked **V1382**/dithiol interlayer, the interfacial recombination could be suppressed. As confirmed by impedance spectroscopy (Figure S21), the parallel resistance of the device increased from 71 to 252 Ω cm^2^ after inserting the cross-linked **V1382**/dithiol interlayer, supporting the above statement. The operational stability of the devices was assessed by running them at the maximum power point under AM 1.5G for 24 h. As shown in Figure 4f, the PCE of the reference device degraded to 60% of its initial value after 16 h, while the device with the cross-linked interlayer still maintained 84% of its initial output after 24 h. In addition, a thermal durability test on the unencapsulated devices was also carried out under an ambient atmosphere. The results are listed in Figure S22. After heating the devices at 100 °C for 1 h, the efficiency of the reference device without the interlayer dropped to 58% of the initial efficiency. In contrast, under the same conditions, the efficiency of the device using the cross-linked **V1382**/dithiol interlayer still retained 71%. Since the cross-linked **V1382**/dithiol with a water contact angle of 69° (Figure S23) shows similar hydrophobicity to doped **Spiro-OMeTAD**,^57,58^ the better stability of the cross-linked **V1382**/dithiol-based PSCs could be attributed to the suppression of the metal diffusion^59^ and the interfacial defect passivation,^60,61^ caused by the insertion of the sulfur-rich interlayer.

**Figure 5:**
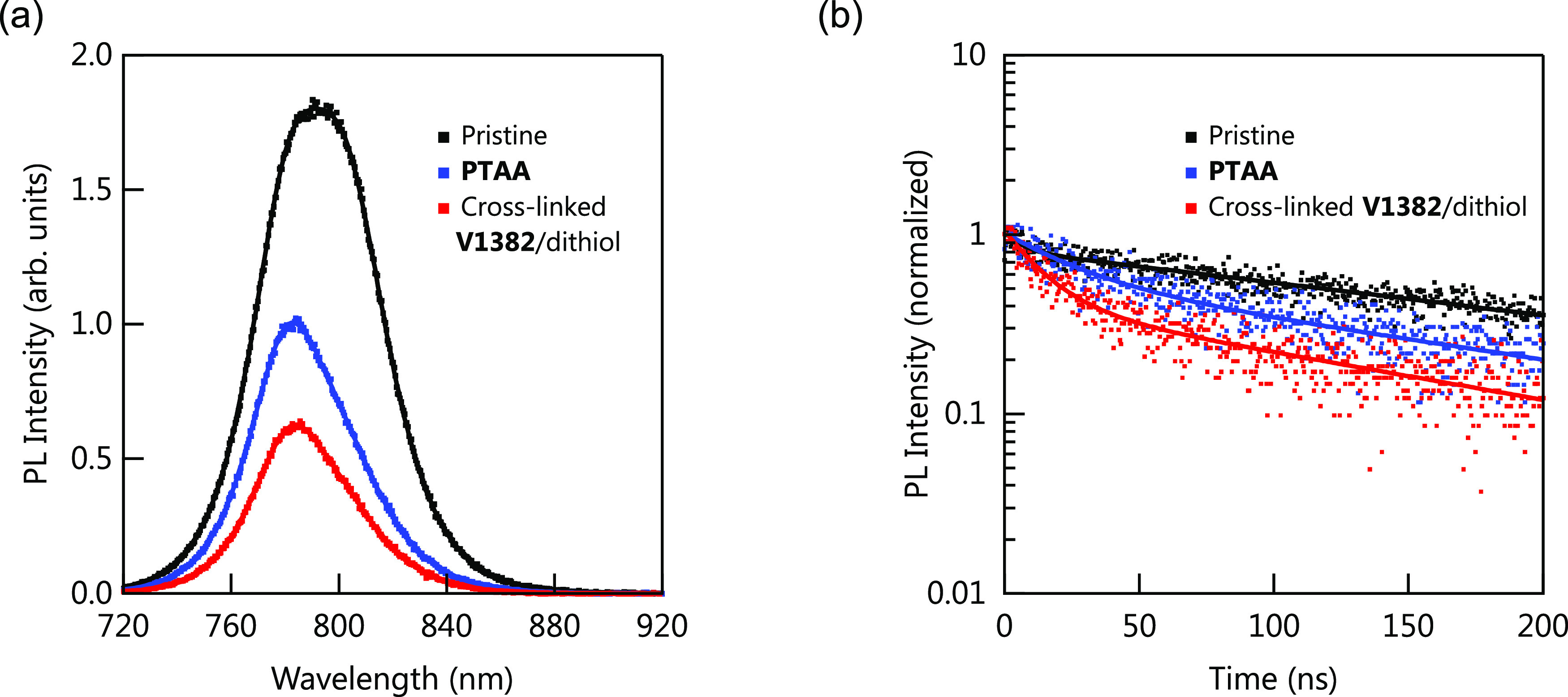
(a) Steady-state PL and (b) time-resolved PL spectra of the perovskite films (Cs_0.05_FA_0.80_MA_0.15_PbI_2.75_Br_0.25_) fabricated on quartz, **PTAA**, and cross-linked **V1382**/dithiol substrates excited at 688 nm with an excitation fluence of 100 nJ cm^–2^. The perovskite is probed through the glass side.

In order to investigate the interfacial charge transfer kinetics, steady-state photoluminescence (PL) quenching and time-resolved PL (TRPL) decay on the perovskite films deposited on quartz, **PTAA**, and cross-linked **V1382**/dithiol were conducted.^62^ As shown in Figure 5a, after fabricating perovskite on HTM layers, the PL peak intensity was reduced, falling to 56 and 35% for **PTAA** and cross-linked **V1382**/dithiol, respectively. The TRPL lifetime for the pristine perovskite film was found to be 196 ns, and the TRPL lifetime for HTM/perovskite films decreased to 120 and 75 ns for **PTAA** and cross-linked **V1382**/dithiol, respectively (Figure 5b). The stronger PL quenching together with the shorter PL lifetime indicates that cross-linked **V1382**/dithiol has a better hole extraction ability than **PTAA**. There is no significant difference between the PL properties of perovskite/**Spiro-OMeTAD** and perovskite/cross-linked **V1382** interlayer/**Spiro-OMeTAD** (Figure S24 and Table S4).

## Conclusions

In summary, a low-cost 9,9′-spirobifluorene derivative bearing four vinyl groups (**V1382**) was designed and synthesized. Due to the presence of vinyl groups, **V1382** can undergo thermal cross-linking at 255 °C to form a solvent-resistant polymeric network. Importantly, by mixing **V1382** with 4,4′-thiobisbenzenethiol in a molar ratio of 1:2, the cross-linking temperature can occur at 103 °C via a facile thiol–ene reaction. The cross-linked **V1382**/dithiol film exhibits appropriate hole mobility and ionization potential, implying its potential as an HTM in PSCs. Taking advantage of the low cross-linking temperature, the cross-linked **V1382**/dithiol can be used as the HTM and HTM interlayer in p–i–n and n–i–p PSC devices, respectively. Devices with the cross-linked **V1382**/dithiol were found to show suppressed interfacial recombination, resulting in better power conversion efficiencies and operational stability than devices using conventional hole-transporting materials such as **PTAA** and **Spiro-OMeTAD**.

## Materials and Methods

### Fabrication of p–i–n PSCs

#### Preparation of Transparent Conductive Oxide Substrates

Glass/FTO substrates (10 sq^–1^, AGC, Inc.) were etched with zinc powder and HCl (6 M in deionized water) and consecutively cleaned with 15 min ultrasonic bath in water, acetone, detergent solution (Semico Clean 56, Furuuchi chemical), water, and isopropanol, followed by drying with an air gun, and finally plasma treatment. The substrates were transferred to an inert gas-filled glovebox for further processing.

#### Preparation of Hole-Transporting Layers

**V1382** was mixed with 4,4′-thiobisbenzenethiol (molar ratio = 1:2, concentration of **V1382** = 0.125–4 mg mL^–1^) in chlorobenzene. The HTM solution (100 μL) was deposited on the FTO substrate using spin-coating (3000 rpm for 30 s, 5 s acceleration), followed by heating on a hot plate at 110 °C for 1 h. In the case of bare **V1382**, 8 mg mL^–1^**V1382** was used. The hole-collecting material PTAA (2.0 mg mL^–1^ in anhydrous toluene) was deposited by using spin-coating (4000 rpm for 30 s, 5 s acceleration), followed by heating on a hot plate at 100 °C for 10 min.

#### Preparation of Perovskite Layer

The Cs_0.05_FA_0.80_MA_0.15_PbI_2.75_Br_0.25_ precursor solution was prepared from CsI (69 mg, 0.27 mmol), MABr (85 mg, 0.76 mmol), PbI_2_ (2.24 g, 4.85 mmol), PbBr_2_ (96 mg, 0.26 mmol), and FAI (703 mg, 4.09 mmol) dissolved in a mixture of DMF (3.0 mL) and DMSO (0.90 mL). After stirring at 40 °C for 30 min, the solution was filtered with a 0.45 μm PTFE filter. 190 μL of the solution was placed on an FTO/HTM substrate and spread by spin-coating (slope 1 s, 1000 rpm 10 s, slope 5 s, 6000 rpm 20 s, slope 1 s) to make a thin film. 300 μL of chlorobenzene was dripped over the rotating substrate at 3 s before the end of the spinning at 6000 rpm. The films were then annealed on a hot plate at 150 °C for 10 min. These perovskite samples were moved under Ar to a vacuum deposition chamber, where 0.5 nm of ethylenediammonium diiodide (EDAI_2_) (deposition rate 0.03 nm s^–1^) was deposited by thermal evaporation.

#### Preparation of Electron-Transporting Layer and Metal Electrode

The above samples were moved under Ar to a vacuum deposition chamber, where 20 nm of C_60_ (deposition rate 0.05 nm s^–1^) and 8 nm of BCP (deposition rate 0.01 nm s^–1^) were deposited by thermal evaporation. The top electrode was prepared by depositing 100 nm of silver (deposition rate, 0.005 nm s^–1^) through a shadow mask.

### Fabrication of n–i–p PSCs

#### Preparation of Transparent Conductive Oxide Substrates

Glass/ITO substrates (10 sq^–1^) were etched with zinc powder and HCl (6 M in deionized water) and consecutively cleaned with a 15 min ultrasonic bath in water, acetone, detergent solution (Semico Clean 56, Furuuchi chemical), water, and isopropanol, followed by drying with an air gun, and finally plasma treatment. The substrates were transferred to an inert gas-filled glovebox for further processing.

#### Preparation of the SnO_2_ Layer

The SnO_2_ layer was prepared by spin-coating a colloidal dispersion (15% in H_2_O) diluted with deionized water (volume ratio = 1:1) on the ITO substrates (400 μL for each substrate, slope 2 s, 3000 rpm 20 s, slope 2 s) followed by annealing at 150 °C for 30 min. A plasma treatment was performed after cooling the substrate to room temperature, before transferring the samples to an inert gas-filled glovebox for further processing.

#### Preparation of Perovskite Layer

The Cs_0.05_FA_0.80_MA_0.15_PbI_2.75_Br_0.25_ precursor solution was prepared from CsI (69 mg, 0.27 mmol), MABr (85 mg, 0.76 mmol), PbI_2_ (2.24 g, 4.85 mmol), PbBr_2_ (96 mg, 0.26 mmol), and FAI (703 mg, 4.09 mmol) dissolved in a mixture of DMF (3.0 mL) and DMSO (0.90 mL). After stirring at 40 °C for 30 min, the solution was filtered with a 0.45 μm PTFE filter. 190 μL of the solution was placed on a glass/ITO/SnO_2_ substrate and spread by spin-coating (slope 1 s, 1000 rpm 10 s, slope 5 s, 6000 rpm 20 s, slope 1 s) to make a thin film. 300 μL of chlorobenzene was dripped over the rotating substrate at 3 s before the end of the spinning at 6000 rpm. The films were then annealed on a hot plate at 150 °C for 10 min.

#### Preparation of Cross-Linked V1382 Interlayer

**V1382** was mixed with 4,4′-thiobisbenzenethiol (molar ratio = 1:2, concentration of **V1382** = 1.0, 2.0 mg mL^–1^) in chlorobenzene. 100 μL of the solution was spin-coated on top of the perovskite layer (3000 rpm for 30 s, 5 s acceleration), followed by heating on a hot plate at 110 °C for 1 h.

#### Preparation of Hole-Transporting Layer

Spiro-OMeTAD (0.06 M) was mixed with an oxidizing agent [tris(2-(1*H*-pyrazol-1-yl)-4-*tert-*butylpyridine)cobalt(III) tris(bis(trifluoromethylsulfonyl)imide)] (FK209, 0.15 equiv) into a solution of chlorobenzene, 4-*tert*-butylpyridine (*t*BP, 3.3 equiv), and lithium bis(trifluoromethylsulfonyl)imide (LITFSI, 0.54 equiv). After being stirred at 70 °C for 30 min, the suspension was filtered with a 0.45 μm PTFE filter to remove insoluble Co(II) complexes. 90 μL of the solution was spin-coated on top of **V1382** (slope 4 s, 4000 rpm, 30 s, slope 4 s), followed by annealing at 70 °C for 30 min.

#### Preparation of Metal Electrode

Gold electrodes (80 nm) were thermally deposited on the top face of the devices by using a shadow mask.

## Supporting Information Available

Equipment and characterization; detailed synthetic procedures, DSC, UV–vis, PESA, XPS, and SEM data; detailed photovoltaic parameters along with *J–V* curves; and PL data (PDF)
