# Supplementary material for: In Situ Thermal Cross-Linking of 9,9′-Spirobifluorene-Based Hole-Transporting Layer for Perovskite Solar Cells
Source: ACS Appl Mater Interfaces. 2023 Dec 20;16(1):1206–16. doi: 10.1021/acsami.3c13950 (PMC10788832; doi:10.1021/acsami.3c13950)
Supplement: Supplementary file 1 — am3c13950_si_001.pdf [file am3c13950_si_001.pdf]

Supporting Information

In Situ Thermal Cross-Linking of 9,9′-Spirobifluorene-Based Hole-Transporting Layer for Perovskite Solar Cells

Sarune Daskeviciute-Geguziene,[‡,a] *Minh Anh Truong,*[‡,b] *Kasparas Rakstys,*[a] *Maryte Daskeviciene,*[a] *Ruito Hashimoto,*[b] *Richard Murdey,*[b] *Takumi Yamada,*[b] *Yoshihiko Kanemitsu,*[b] *Vygintas Jankauskas,*[c] *Atsushi Wakamiya,**[*,b] *Vytautas Getautis*[*,a]

[a]Department of Organic Chemistry, Kaunas University of Technology, Radvilenu pl. 19, Kaunas 50254, Lithuania.

[b]Institute for Chemical Research, Kyoto University, Gokasho, Uji, Kyoto 611-0011, Japan.

[c]Institute of Chemical Physics, Vilnius University, Sauletekio al. 3, Vilnius 10257, Lithuania.

‡S.D.-G. and M.A.T. contributed equally to this work.

*Corresponding Authors:

wakamiya@scl.kyoto-u.ac.jp; vytautas.getautis@ktu.lt

EXPERIMENTAL SECTION

Equipment and Characterization

Chemicals were purchased from Sigma-Aldrich, TCI Europe and used as received without further purification.

^{1}H NMR spectra were recorded at 400 MHz on a Bruker Avance III spectrometer with a 5 mm double resonance broad band BBO *z*-gradient room temperature probe, ^{13}C NMR spectra were collected using the same instrument at 101 MHz. The chemical shifts, expressed in ppm, were relative to tetramethylsilane (TMS). All the NMR experiments were performed at 25 °C. Reactions were monitored by thin-layer chromatography on ALUGRAM SIL G/UV254 plates and developed with UV light. Silica gel (grade 9385, 230–400 mesh, 60 Å, Aldrich) was used for column chromatography.

Elemental analysis (EA) was performed with an Exeter Analytical CE-440 elemental analyser, Model 440 C/H/N/.

Mass spectrometry (MS) was performed on Waters SQ Detector 2 Spectrometer using electrospray ionization (ESI) technique.

Thermogravimetric analysis (TGA) was performed on a Q50 thermogravimetric analyser at a scan rate of 10 ºC min^{-1} under a nitrogen atmosphere. The values are given for a weight loss of 5%.

UV–Vis spectral analysis of the samples solutions (THF, 10^{-4} M) was performed on a Perkin Elmer Lambda 35 UV–Vis spectrophotometer. The layer thickness of the solution is d=1 mm. Diffraction grating crack width 2 nm. Spectral recording speed 2 nm s^{-1}. The wavelength λ is given in nm. The time-resolved fluorescence spectra of the samples solutions (THF, 10^{-5} M) were recorded on a Edinburgh Instruments FLS920 light emission intensity spectrophotometer. Solution of new compound exposed to wavelength 390 nm. The layer thickness of the solution is d=1 cm. The wavelength λ is given in nm.

FT-IR spectra were recorded by using a Perkin–Elmer Frontier spectrophotometer with a single reflectance horizontal ATR (Attenuated Total Reflectance) cell equipped with a diamond crystal. The data were recorded in the spectral range from 655 to 4000 cm^{-1} by accumulating 5 scans with a resolution of 4 cm^{-1}.

The samples for the ionization potential I_p measurement were prepared by dissolving materials in THF and were coated on Al plates pre-coated with ~0.5μm thick methylmethacrylate and methacrylic acid copolymer adhesive layer. The thickness of the HTM layer was 0.5–1 μm. The samples were illuminated with monochromatic light from the quartz monochromator with deuterium lamp. The power of the incident light beam was (2–5)×10^{-8} W. The negative voltage of −300 V was supplied to the sample substrate. The counter-electrode with the 4.5 mm×15 mm slit for illumination was placed at 8 mm distance from the sample surface. The counter-electrode was connected to the input of the BK2-16 type electrometer, working in the open input regime, for the photocurrent measurement. The 10^{-15} to 10^{-12} A strong photocurrent was flowing in the circuit under illumination. The samples for mobility measurements were prepared from the neat material. The sample substrate was glass plates with conductive Al layer. The layer thickness was in the range of 3.5–5 μm.

The hole drift mobility was measured by xerographic time of flight (XTOF) technique. Positive corona charging created electric field inside the HTM layer. Charge carriers were generated at the layer surface by illumination with pulses of N_2 laser (pulse duration was 1 ns, wavelength 337 nm).

Scanning electron microscopy (SEM) was performed with a Hitachi S8010 ultra-high-resolution scanning electron microscope (Hitachi High-Tech Corporation).

For the time-resolved photoluminescene (TRPL) measurements, the samples were excited by a picosecond pulsed light with a wavelength of 688 nm (Advanced Laser Diode System). The excitation fluence was set at 100 nJ cm^{-2}. The PL signals were recorded using an avalanche photodiode (ID Quantique) and a time-correlated single photon counting board (PicoQuant). The PL lifetimes were obtained by fitting the PL decay curve with a double exponential function and calculating the average lifetime. The PL spectra were recorded using a N_2 cooled charge-coupled-device array equipped with a monochromator (Princeton Instruments). The samples were kept in an Ar-filled metallic box for the whole process to avoid oxygen contamination and degradation.

X-ray photoelectron spectroscopy (XPS) was recorded with a JPS-9010 (JEOL Co., Ltd.) instrument. Perovskite film samples for XPS measurements were prepared in a N_2-filled glove box and transferred to the XPS chamber through a N_2-filled transfer vessel in order to avoid contamination.

Photocurrent–voltage (*J–V*) measurements for perovskite solar cells were measured in air with an OTENTO-SUNIII (BUNKOUKEIKI Co., Ltd.). The light intensity of the illumination source was adjusted by using standard silicon photodiodes (BS520). Each device was measured with a 20 mV voltage step and a 200 ms time step (i.e. scan rate of 0.1 V s^{-1}) using a Keithley 2400 source meter.

The device active area was defined by an optimal mask (0.1 cm^2). Steady-state power output (SPO) measurements were performed by holding the device at the voltage of the maximum power point, as determined by the JV characteristic, and monitoring the current density over the course of 1000 s.

External quantum efficiency (EQE) and internal quantum efficiency (IQE) spectra were measured with a Bunkoukeiki SMO-250III system equipped with a Bunkoukeiki SM-250 diffuse reflection unit (Bunkoukeiki Co., Ltd.). The incident light intensity was calibrated with a standard SiPD S1337-1010BQ silicon photodiode.

Impedance spectroscopy data was obtained in air with a 4192 LF impedance analyzer (Hewlett-Packard Company), at zero volts applied bias, 30 mV oscillator voltage, 20–200,000 Hz frequency scan, with the measurement devices under AM 1.5G simulated solar radiation with a 0.1 cm^{-2} shadow mask. The impedance data was fit by using the following model circuit:

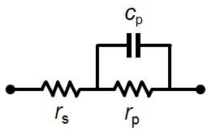

Synthesis

Scheme S1. Synthetic route of the **V1382**.

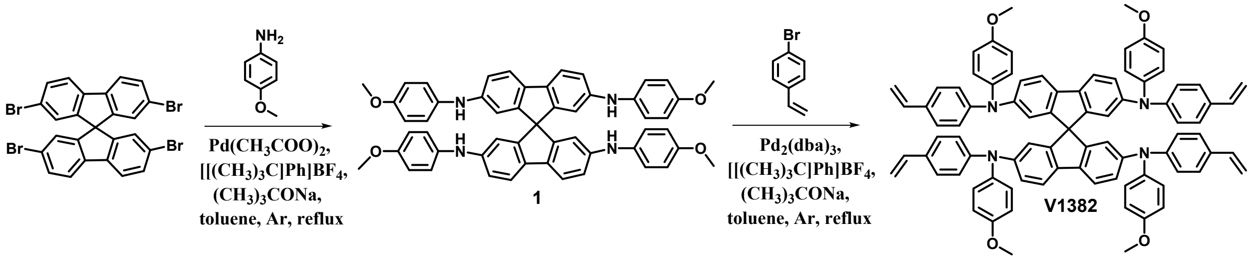

***N^2^*,*N^2'^*,*N^7^*,*N^7'^*-tetrakis(4-methoxyphenyl)-9,9'-spirobi[fluorene]-2,2',7,7'-tetraamine (1)**

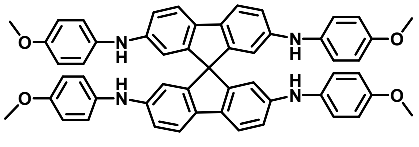

1

A solution of compound 2,2',7,7'-Tetrabromo-9,9'-spirobifluorene (3 g, 4.7 mmol, 1 equiv) and *p*-anisidine (5.9 g, 47.5 mmol, 10 equiv) in anhydrous toluene (47 mL) was purged with argon for 30 minutes. Afterwards, palladium (II) acetate (0.02 equiv), tri-*tert*-butylphosphonium tetrafluoroborate (0.027 equiv) and sodium *tert*-butoxide (8 equiv) were added and the resulted solution was refluxed under argon atmosphere for 28 hours. After cooling down to room temperature, the reaction mixture was extracted with ethyl acetate and distilled water. The organic layer was dried over anhydrous Na_2SO_4, filtered and solvent evaporated. The crude product was purified by column chromatography using 8:17 v/v THF/*n*-hexane as an eluent. Pale green solid were collected as a final product. (2.7 g, 71% yield). ^{1}H NMR (400 MHz, DMSO-d_6) δ 7.73 (s, 4H), 7.54 (d, J = 8.0 Hz, 4H), 7.00 – 6.87 (m, 12H), 6.79 (d, J = 8.4 Hz, 8H), 6.23 (s, 4H), 3.67 (s, 12H). ^{13}C NMR (101 MHz, DMSO-d_6) δ 154.14, 150.79, 144.28, 136.61, 132.96, 120.61, 119.86, 114.96, 113.37, 111.64, 65.48, 55.65 ppm. Anal. calcd for $C_{53}H_{44}N_4O_4$: C, 79.48; H, 5.54; N, 7.00; found: C, 79.77; H, 5.52; N, 7.04.

***N*^{2},*N*$^{2'}$,*N*^{7},*N*$^{7'}$-tetrakis(4-methoxyphenyl)-*N*^{2},*N*$^{2'}$,*N*^{7},*N*$^{7'}$-tetrakis(4-vinylphenyl)-9,9'-spirobi[fluorene]- 2,2',7,7'-tetraamine (V1382)**

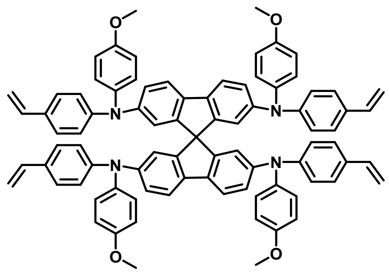

V1382

A solution of compound **1** (0.2 g, 0.2 mmol, 1 equiv) and 4-bromostyrene (0.2 g, 1.1 mmol, 4.5 equiv) in anhydrous toluene (22 mL) was purged with argon for 30 minutes. Afterwards, tris(dibenzylideneacetone)dipalladium(0) (0.063 equiv), tri-*tert*-butylphosphonium tetrafluoroborate (0.09 equiv) and sodium *tert*-butoxide (6 equiv) were added and the resulted solution was refluxed under argon atmosphere for 5 hours. After cooling down to room temperature, the reaction mixture was filtered through celite, extracted with ethylacetate and distilled water. The organic layer was dried over anhydrous Na_2SO_4, filtered and solvent evaporated. The crude product was purified by column chromatography using 1:4 v/v THF/*n*-hexane as an eluent. The obtained product was precipitated from THF into 15 times excess of ethanol. The precipitate was filtered off and washed with ethanol to collect **V1382** as a pale yellow solid. (0.15 g, 51% yield). ^{1}H NMR (400 MHz, THF-d_6) δ 7.51 (d, J = 8.4 Hz, 4H), 7.20 (d, J = 8.6 Hz, 8H), 6.97 (d, J = 8.6 Hz, 8H), 6.92 – 6.78 (m, 20H), 6.67 – 6.53 (m, 8H), 5.57 (d, J = 17.6 Hz, 4H), 5.05 (d, J = 10.9 Hz, 4H), 3.75 (s, 12H). ^{13}C NMR (101 MHz, THF-d_6) δ 154.68, 148.14, 146.11, 144.97, 138.45, 134.54, 134.47, 128.93, 124.82, 124.81, 122.14, 119.29, 118.32, 117.12, 112.68, 108.59, 63.78, 52.76 ppm. Anal. calcd for $C_{85}H_{68}N_4O_4$: C, 84.41; H, 5.67; N, 4.63; found: C, 84.27; H, 5.62; N, 4.65. $C_{85}H_{68}N_4O_4[M^+]$ exact mass = 1208.52, MS (ESI) = 1208.61.

Table S1. Materials, Quantities, and Cost for the Synthesis of **V1382**

Chemical	Weight reagent (g/g)	Weight solvent (g/g)	Weight workup (g/g)	Price of chemical (€/kg)	Cost of chemical (€/g product)	Total cost (€/g)
2,2',7,7'-Tetrabromo-9,9'-spirobifluorene	1.11			1712	1.90	
p-Anisidine	2.19			219	0.48	
Palladium (II) acetate	0.01			52000	0.52	
Tri-*tert*-butylphosphonium tetrafluoroborate	0.01			755	0.01	
Sodium *tert*-butoxide	1.35			214	0.29	
Toluene		17		2.46	0.04	
Ethyl acetate			200	2.85	0.57	
Na_2SO_4			50	6.08	0.31	
Silicagel			20	64.8	1.30	
THF			470	8.88	4.17	
n-Hexane			1000	8.33	8.33	
***N*^{2},*N*$^{2'}$,*N*^{7},*N*$^{7'}$-tetrakis(4-methoxyphenyl)-9,9'-spirobi[fluorene]-2,2`,7,7'-tetraamine (1)**	4.67	17	1740			17.92
N^2,*N*$^{2'}$,*N*^{7},*N*$^{7'}$-tetrakis(4-methoxyphenyl)-9,9'-spirobi[fluorene]-2,2`,7,7'-tetraamine (1)	1.33			18210	23.83	
4-Bromostyrene	1.37			907	1.24	
Tris(dibenzylideneacetone)dipalladium(0)	0.09			18230	1.64	
Tri-*tert*-butylphosphonium tetrafluoroborate	0.04			755	0.03	
Sodium *tert*-butoxide	0.96			214	0.21	
Toluene		146		2.46	0.36	
Ethyl acetate			200	2.85	0.57	
Celite			20	36.6	0.73	
Na_2SO_4			50	6.08	0.31	
THF			270	8.88	2.40	
n-Hexane			1000	8.33	8.33	
Silicagel			20	64.8	1.30	
Ethanol			100	6.08	0.61	
***N*^{2},*N*$^{2'}$,*N*^{7},*N*$^{7'}$-tetrakis(4-methoxyphenyl)-*N*^{2},*N*$^{2'}$,*N*^{7},*N*$^{7'}$-tetrakis(4-vinylphenyl)-9,9`-spirobi[fluorene]- 2,2`,7,7`-tetraamine (V1382)**	3.79	146	1660			41.56

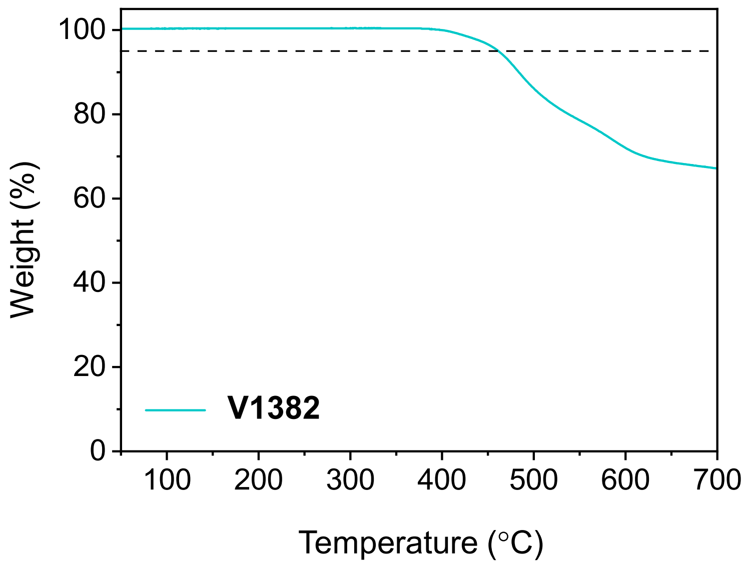

Figure S1. TGA curve of **V1382**.

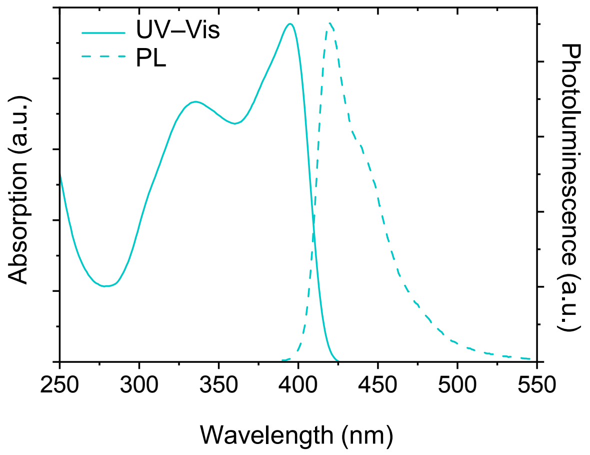

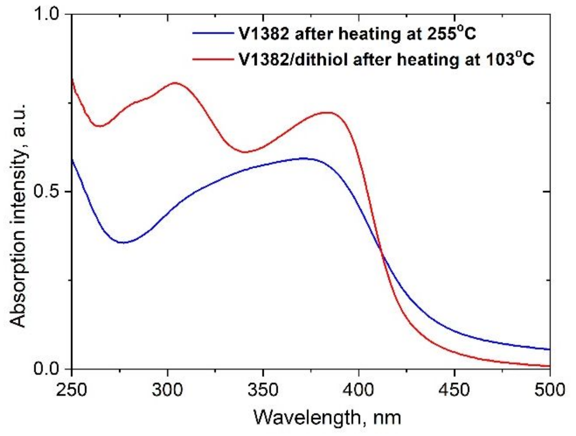

Figure S2. (left) UV–Vis absorption (solid line) and photoluminescence (dashed line) spectra of **V1382** in THF solution (10^{-4} M); (right) UV–Vis absorption of **V1382**-based polymers.

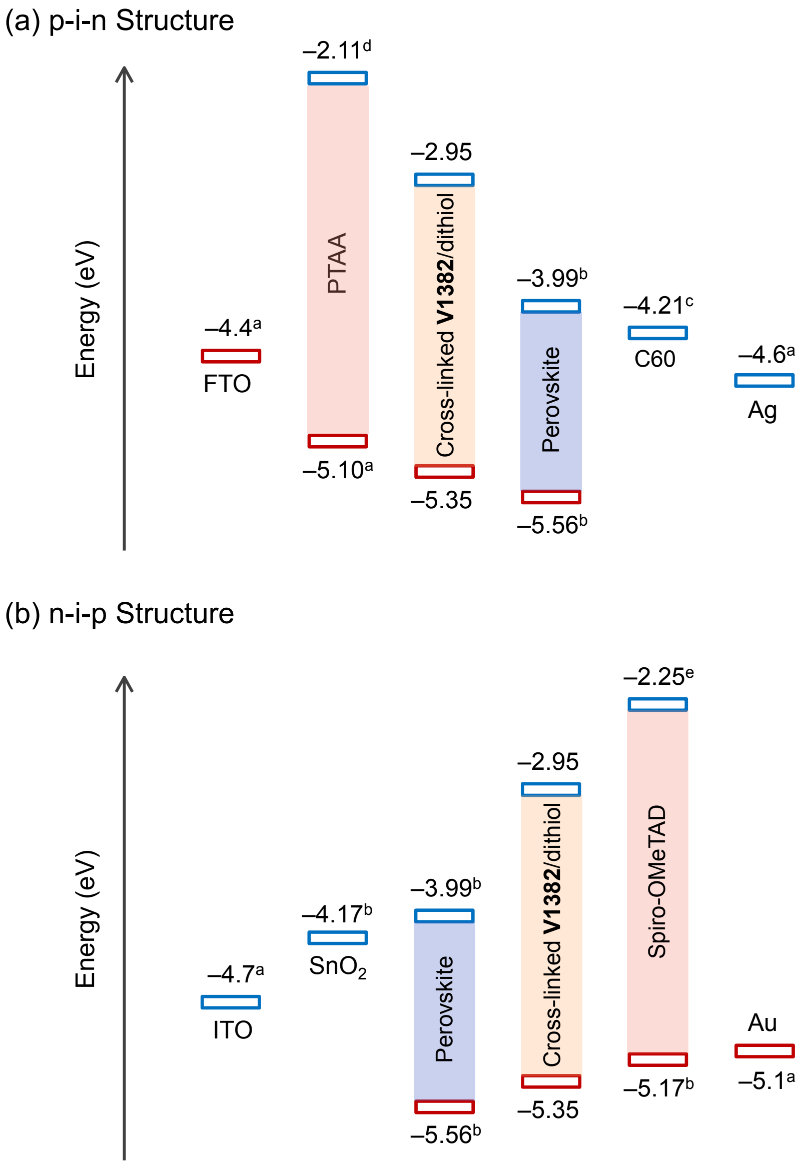

Figure S3. Energy diagrams of (a) p-i-n and (b) n-i-p solar cell devices. [a,b,c]Values taken from the literature [1],[2],[3], respectively; [d]calculated by using optical band gap as reported in [4]; [e]calculated by using optical band gap as reported in [5].

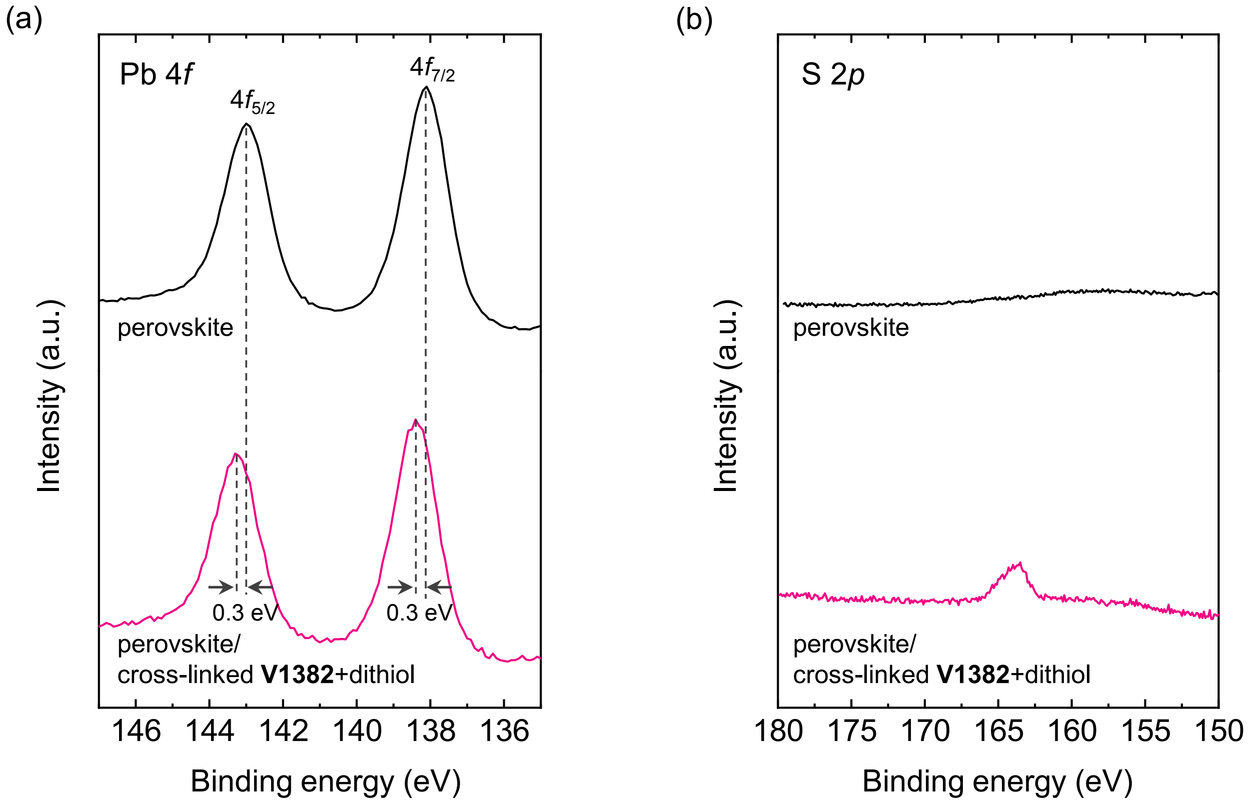

Figure S4. XPS spectra of (a) Pb 4*f* and (b) S 2*p* of the pristine perovskite and perovskite coated with cross-linked **V1382**/dithiol.

Table S2. Photovoltaic Parameters of p-i-n PSCs Fabricated on Bare **V1382** and on Cross-Linked **V1382**/dithiol with Difference Concentration of **V1382** Derived from *J*–*V* Measurements

Concentration[a]	Scan[b]	J_{sc} (mA cm^{-2})[c]	V_{oc} (V)[c]	FF[c]	PCE (%)[c]	HI[d]
bare **V1382** (8 mg mL^{-1})	F	22.5 (22.4 ± 0.2)	1.05 (1.06 ± 0.01)	0.79 (0.78 ± 0.03)	18.5 (18.4 ± 0.4)	0.016
	R	22.3 (22.2 ± 0.1)	1.06 (1.06 ± 0.01)	0.79 (0.75 ± 0.05)	18.8 (17.6 ± 1.1)	
0.125 mg mL^{-1} + dithiol	F	22.2 (22.5 ± 0.2)	1.03 (0.99 ± 0.03)	0.81 (0.80 ± 0.02)	18.6 (17.8 ± 0.7)	–0.409
	R	20.0 (20.2 ± 0.3)	1.01 (1.01 ± 0.01)	0.65 (0.66 ± 0.02)	13.2 (13.5 ± 0.7)	
0.25 mg mL^{-1} + dithiol	F	23.5 (22.5 ± 0.7)	1.04 (1.03 ± 0.01)	0.79 (0.79 ± 0.01)	19.2 (18.3 ± 0.7)	–0.067
	R	22.8 (21.1 ± 1.2)	1.05 (1.02 ± 0.02)	0.75 (0.68 ± 0.04)	18.0 (14.6 ± 1.9)	
0.5 mg mL^{-1} + dithiol	F	22.9 (22.3 ± 1.1)	1.05 (1.03 ± 0.01)	0.80 (0.78 ± 0.02)	19.1 (18.0 ± 0.9)	–0.091
	R	22.2 (21.7 ± 1.1)	1.05 (1.03 ± 0.01)	0.75 (0.70 ± 0.04)	17.5 (15.8 ± 1.7)	
1.0 mg mL^{-1} + dithiol	F	22.5 (21.8 ± 0.6)	1.06 (1.08 ± 0.01)	0.79 (0.78 ± 0.01)	19.0 (18.4 ± 0.5)	–0.145
	R	22.5 (22.1 ± 0.4)	1.03 (1.06 ± 0.02)	0.72 (0.71 ± 0.02)	16.6 (16.5 ± 0.7)	
2.0 mg mL^{-1} + dithiol	F	23.0 (22.6 ± 0.3)	1.09 (1.08 ± 0.01)	0.77 (0.77 ± 0.02)	19.3 (18.7 ± 0.4)	–0.027
	R	23.5 (22.8 ± 0.4)	1.07 (1.07 ± 0.01)	0.75 (0.74 ± 0.02)	18.8 (18.1 ± 0.8)	
4.0 mg mL^{-1} + dithiol	F	23.3 (23.1 ± 0.3)	1.11 (1.09 ± 0.01)	0.67 (0.65 ± 0.02)	17.3 (16.5 ± 0.6)	0.049
	R	23.3 (23.1 ± 0.2)	1.10 (1.09 ± 0.01)	0.71 (0.72 ± 0.01)	18.2 (18.0 ± 0.2)	

[a]HTMs (**V1382**:dithiol/1:2 molar ratio) were spin-coated on FTO substrates from PhCl solution. [b]Forward and reverse indicate the scan direction from J_{SC} to V_{OC} and from V_{OC} to J_{SC}, respectively. [c]The average and standard deviation values were given in parentheses. [d]Hysteresis index (HI) = $(PCE_{Reverse} - PCE_{Forward})/PCE_{Reverse}$.

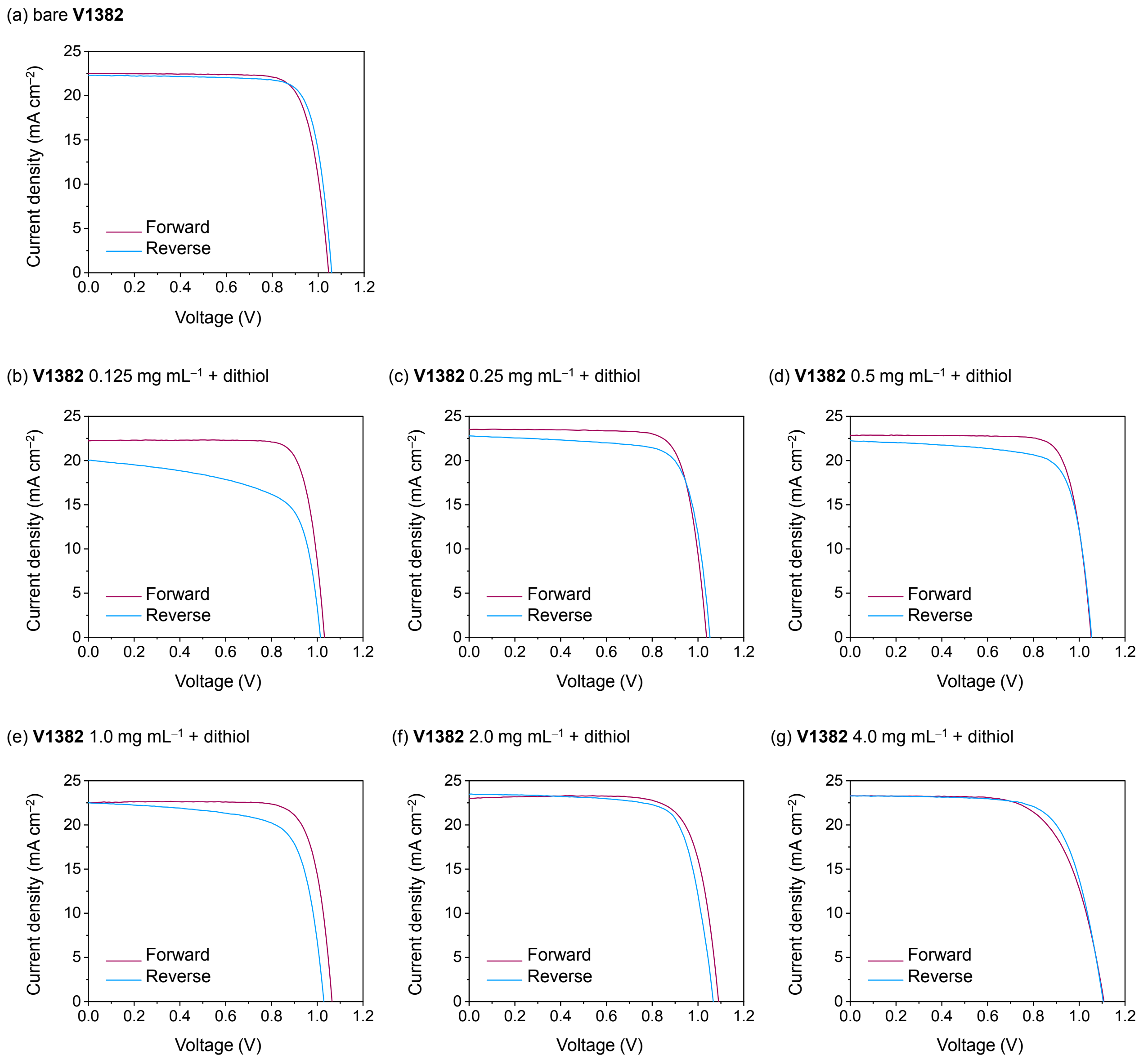

Figure S5. *J*–*V* curves of p-i-n PSCs fabricated on bare (a) **V1382** and on cross-linked (b-g) **V1382**/dithiol with difference concentrations of **V1382**.

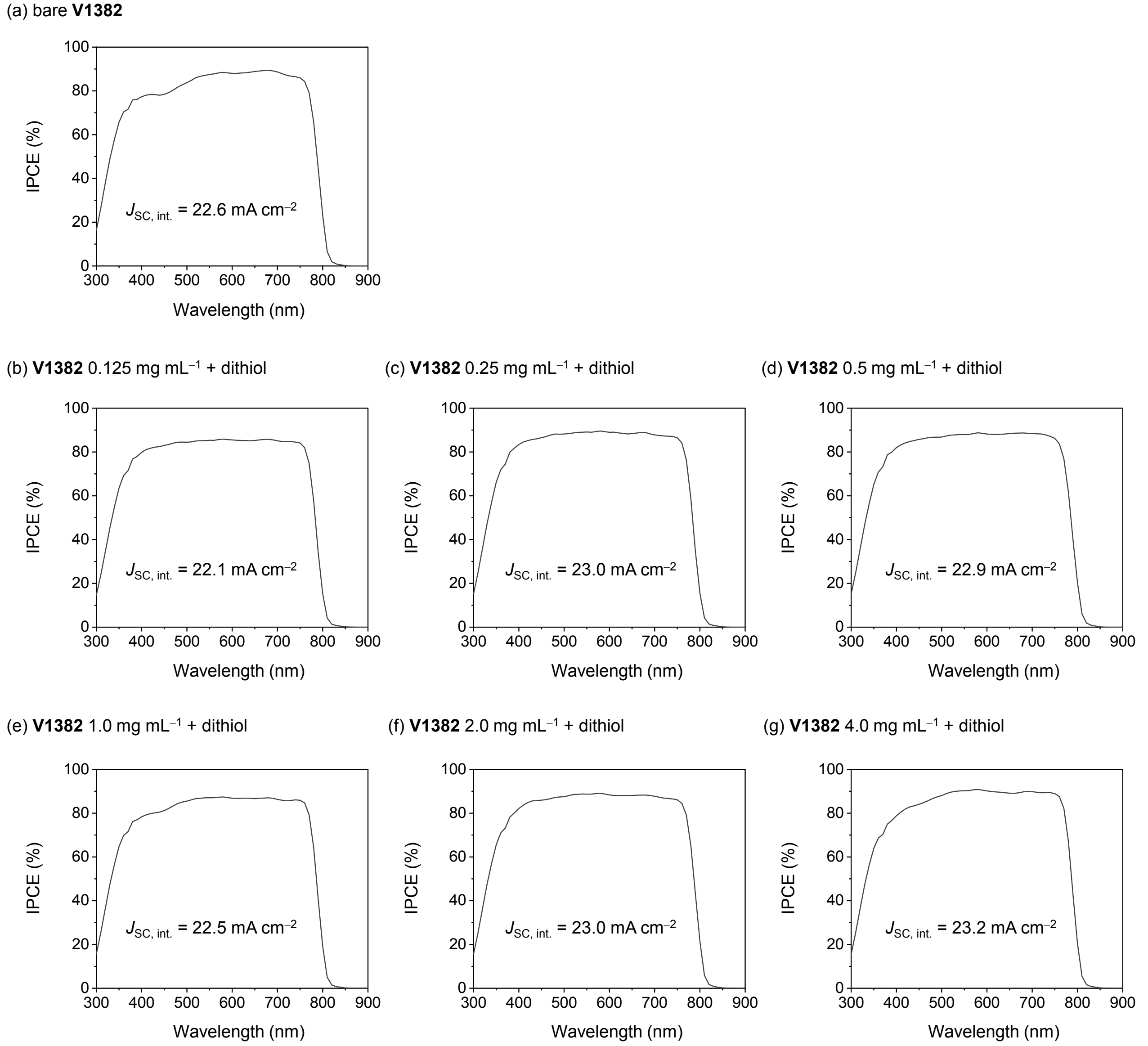

Figure S6. IPCE spectra of p-i-n PSCs fabricated on bare (a) **V1382** and on cross-linked (b-g) **V1382**/dithiol with difference concentrations of **V1382**.

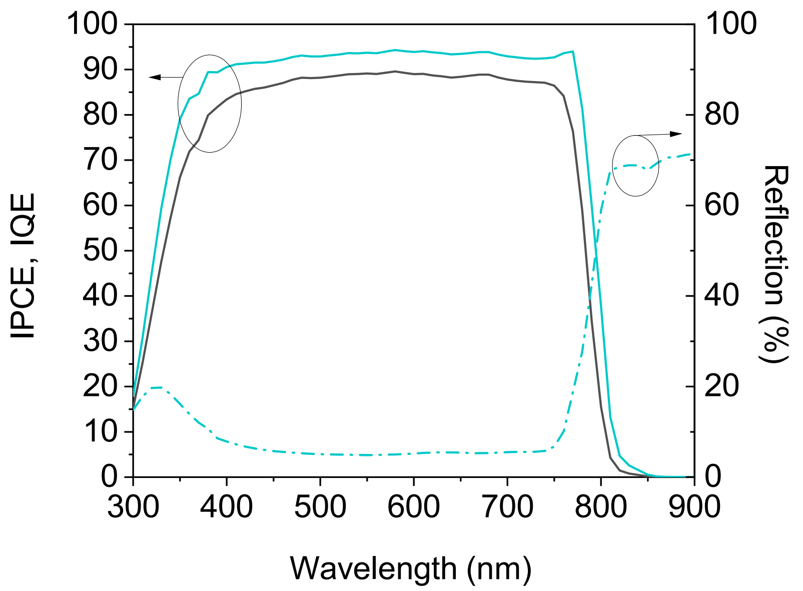

Figure S7. IPCE, IQE, and reflection spectra of p-i-n PSC fabricated on cross-linked **V1382**/dithiol with **V1382** 2.0 mg mL^{-1}.

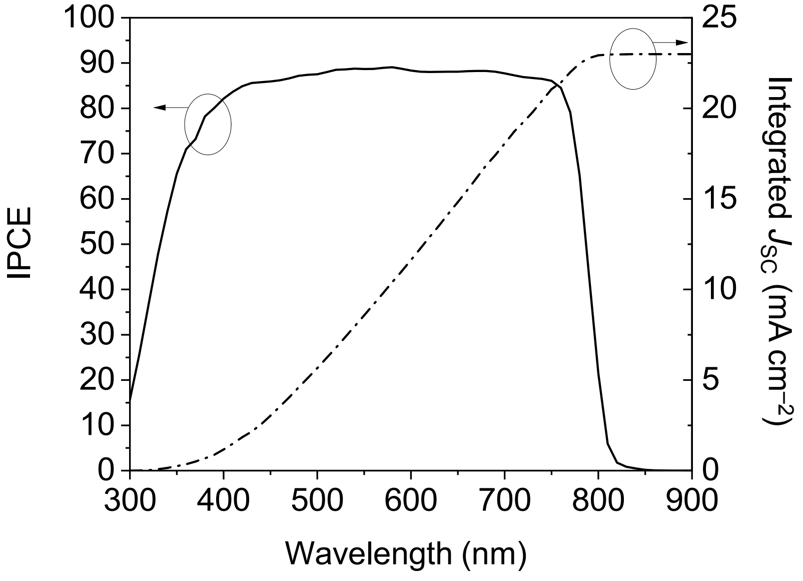

Figure S8. IPCE spectrum and integrated J_{SC} curve of p-i-n PSC fabricated on cross-linked **V1382**/dithiol with **V1382** 2.0 mg mL^{-1}.

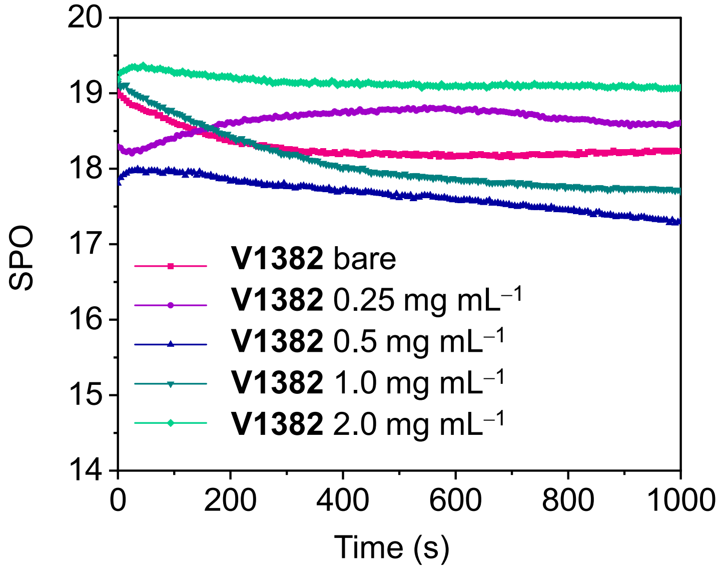

Figure S9. The stabilized power output (SPO) measured under air of unencapsulated p-i-n PSCs fabricated on bare **V1382** and on cross-linked **V1382**/dithiol with difference concentrations of **V1382**.

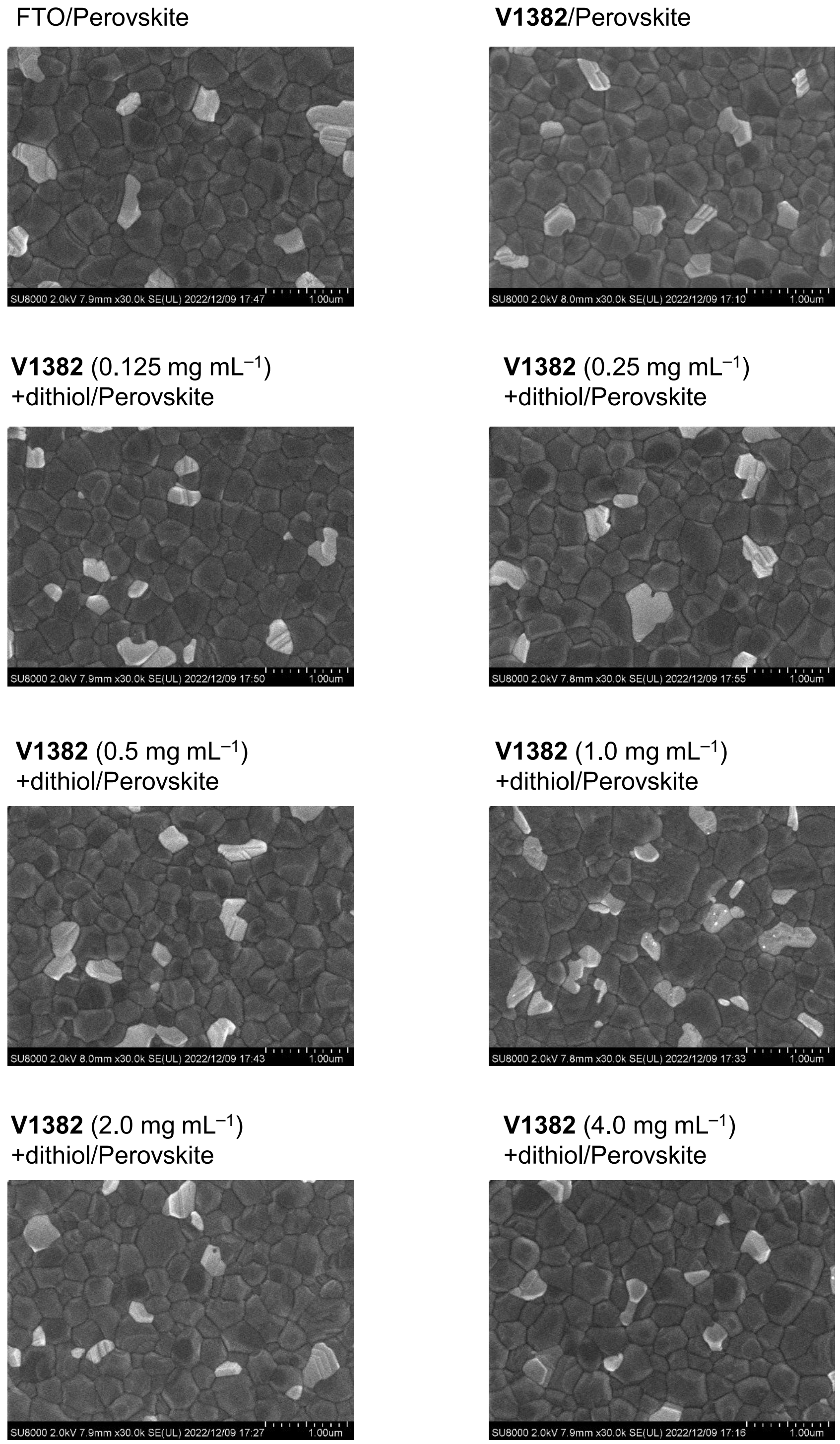

Figure S10. Top-view SEM images of perovskite films deposited on bare **V1382** and on cross-linked **V1382**/dithiol with difference concentrations of **V1382**.

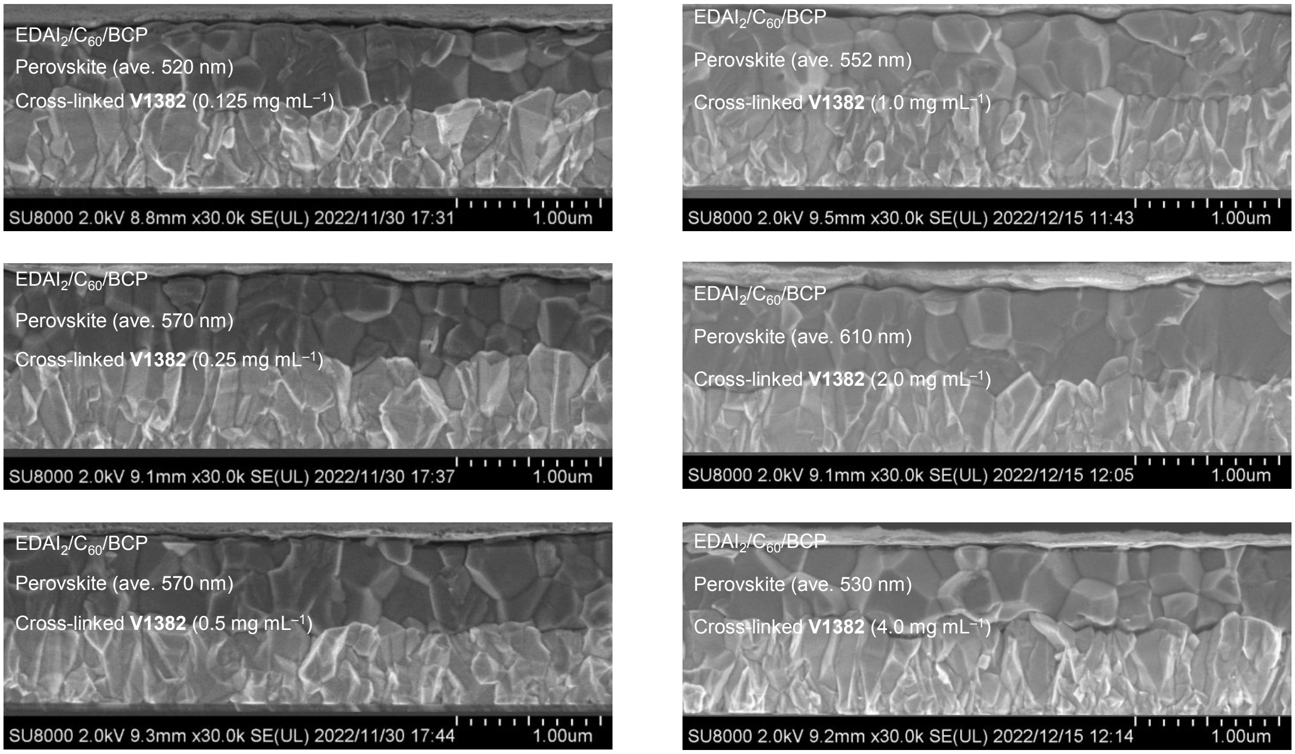

Figure S11. Cross-sectional SEM images of perovskite films fabricated on cross-linked **V1382**/dithiol with difference concentrations of **V1382**.

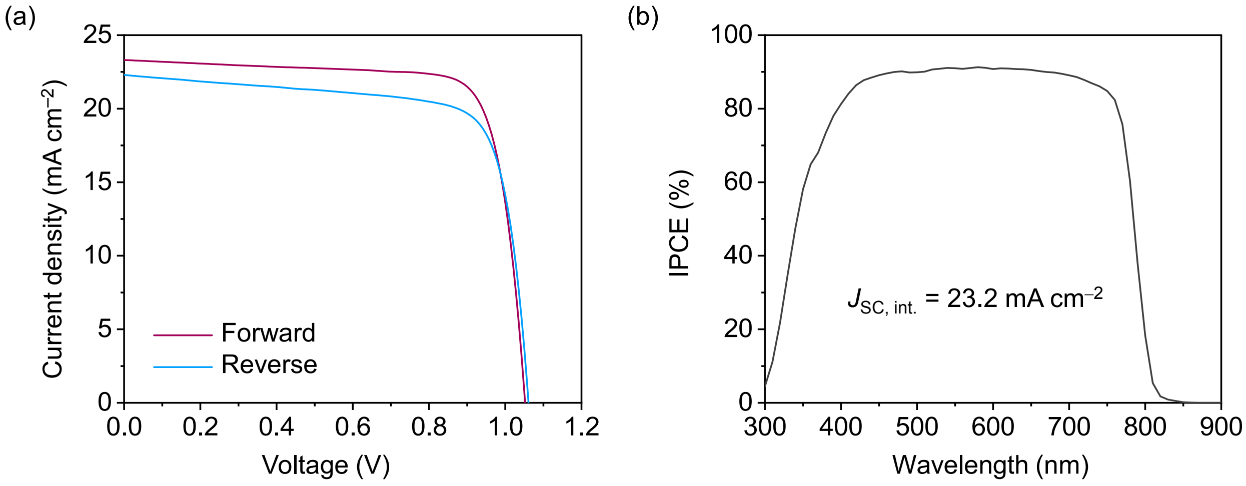

Figure S12. (a) *J*–*V* curves and (b) IPCE spectrum of p-i-n PSC fabricated on PTAA.

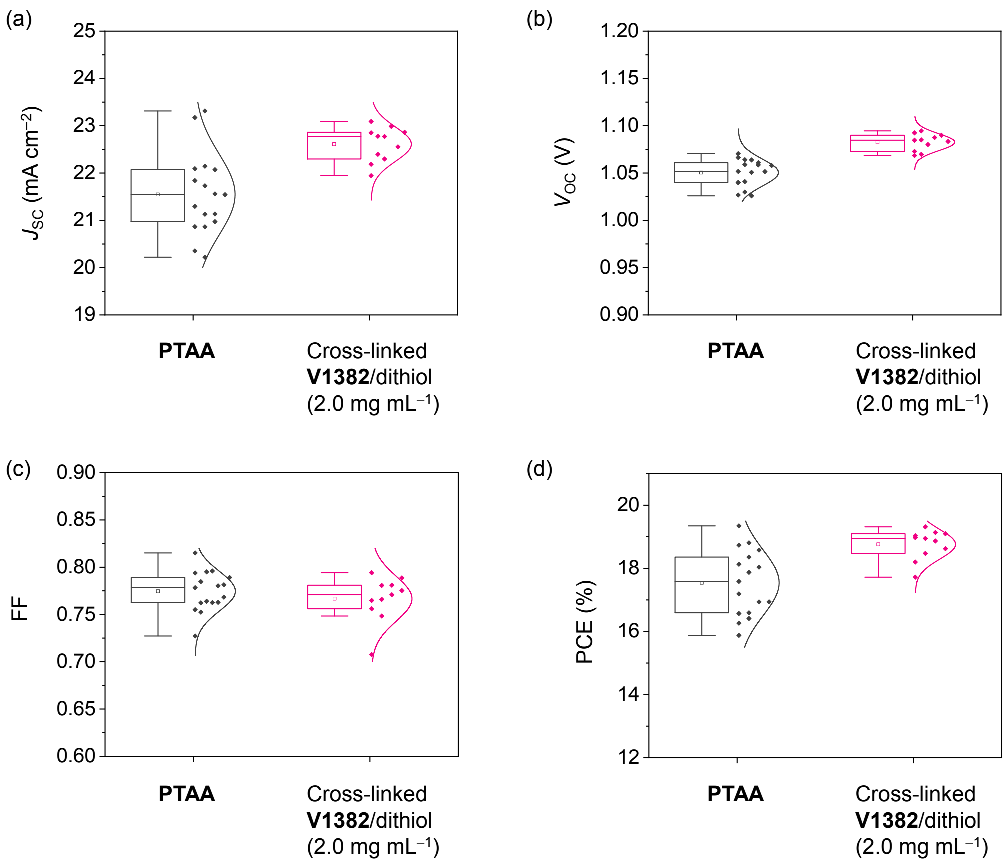

Figure S13. Box plots of (a) J_{SC}, (b) V_{OC}, (c) FF, and (d) PCE of the p-i-n PSCs fabricated by using PTAA and cross-linked **V1382**/dithiol (**V1382** 2.0 mg mL^{-1}) as HTLs obtained in the forward scan.

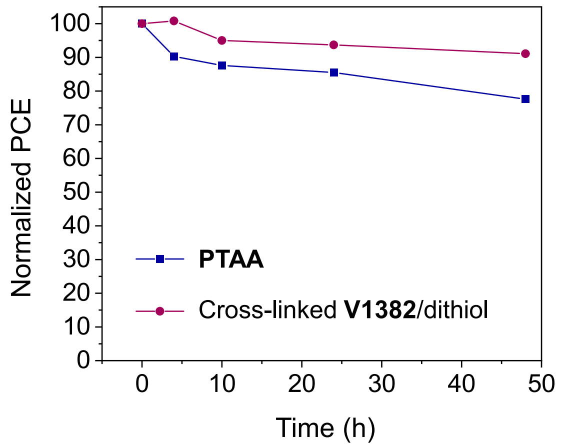

Figure S14. The thermal stability test of the unencapsulated p-i-n PSCs using the cross-linked **V1382**/dithiol and **PTAA** as the HTMs at 85 °C in air with a controlled humidity of 40%. Average data was taken from three devices for each HTM.

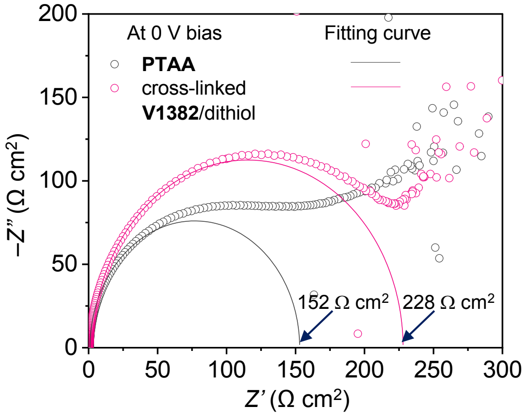

Figure S15. Complex impedance plots of p-i-n PSCs under an inert atmosphere at AM 1.5G illumination and 0 V bias.

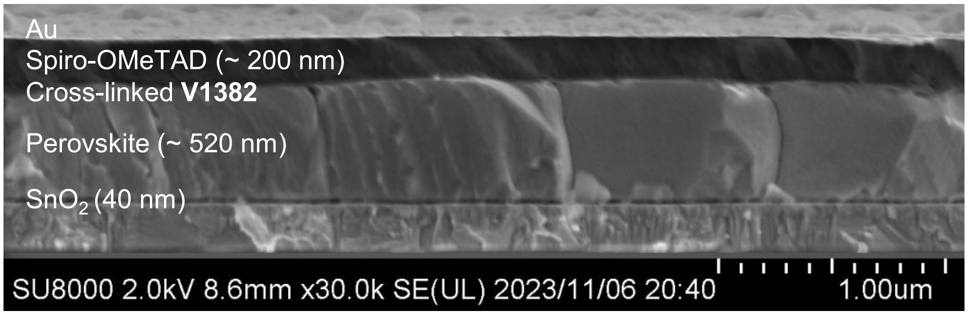

Figure S16. Cross-sectional SEM image of the n-i-p PSC fabricated with cross-linked **V1382**/dithiol as HTM interlayers.

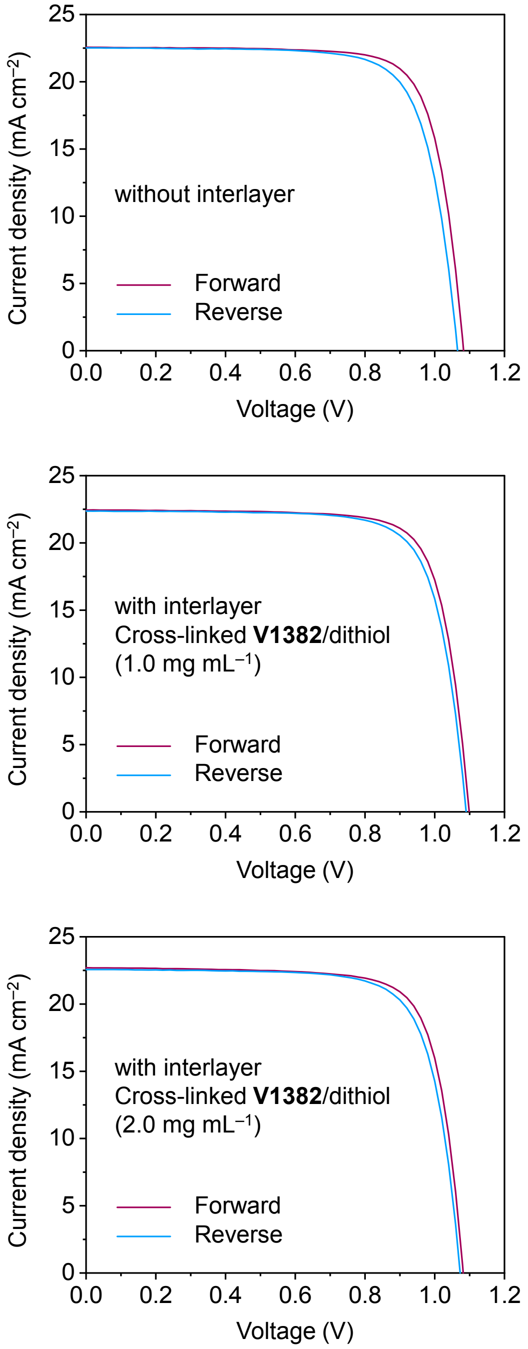

Figure S17. *J*–*V* curves of n-i-p PSCs fabricated without and with cross-linked **V1382**/dithiol as HTM interlayers (with different concentration of **V1382**).

Table S3. Photovoltaic Parameters of n-i-p PSCs Fabricated without and with Cross-Linked **V1382**/dithiol Interlayer Derived from *J–V* Measurements

Concentration[a]	Scan[b]	J_{sc} (mA cm^{-2})[c]	V_{oc} (V)[c]	FF[c]	PCE (%)[c]	HI[d]
without interlayer	F	22.6 (22.2 ± 0.4)	1.08 (1.05 ± 0.02)	0.77 (0.75 ± 0.01)	18.9 (17.5 ± 0.8)	–0.050
	R	22.5 (22.1 ± 0.4)	1.07 (1.06 ± 0.01)	0.75 (0.74 ± 0.01)	18.0 (17.4 ± 0.5)	
1.0 mg mL^{-1} **V1382** + dithiol	F	22.4 (22.1 ± 0.4)	1.10 (1.05 ± 0.03)	0.77 (0.75 ± 0.01)	19.1 (17.6 ± 0.8)	–0.032
	R	22.4 (22.1 ± 0.4)	1.09 (1.06 ± 0.02)	0.76 (0.76 ± 0.01)	18.5 (17.8 ± 0.5)	
2.0 mg mL^{-1} **V1382** + dithiol	F	22.7 (22.3 ± 0.3)	1.08 (1.07 ± 0.01)	0.77 (0.76 ± 0.01)	18.8 (18.2 ± 0.4)	–0.027
	R	22.6 (22.2 ± 0.3)	1.07 (1.07 ± 0.01)	0.75 (0.76 ± 0.01)	18.3 (18.0 ± 0.2)	

[a]HTM interlayers (**V1382**:dithiol/1:2 molar ratio) were spin-coated on top of perovskite layer from PhCl solution. [b]Forward and reverse indicate the scan direction from J_{SC} to V_{OC} and from V_{OC} to J_{SC}, respectively. [c]The average and standard deviation values were given in parentheses. [d]Hysteresis index (HI) = ($PCE_{Reverse}$ –$PCE_{Forward}$)/$PCE_{Reverse}$.

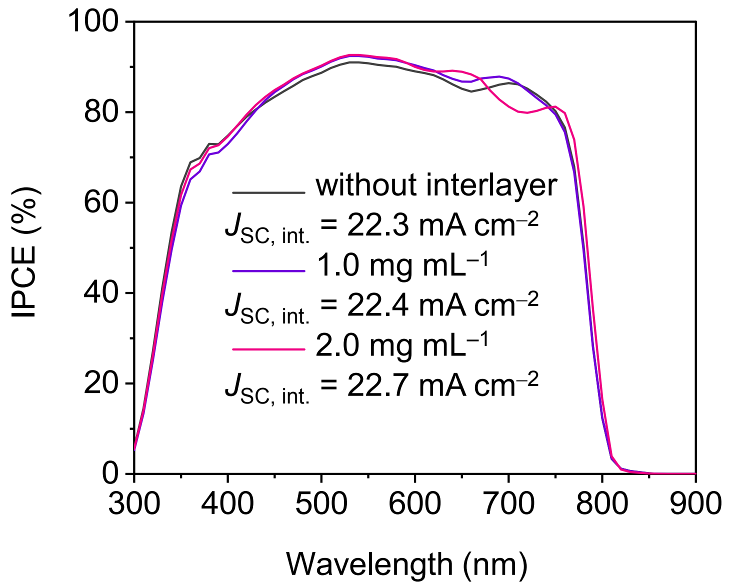

Figure S18. IPCE spectra of n-i-p PSCs fabricated without and with cross-linked **V1382**/dithiol as HTM interlayers (with different concentration of **V1382**).

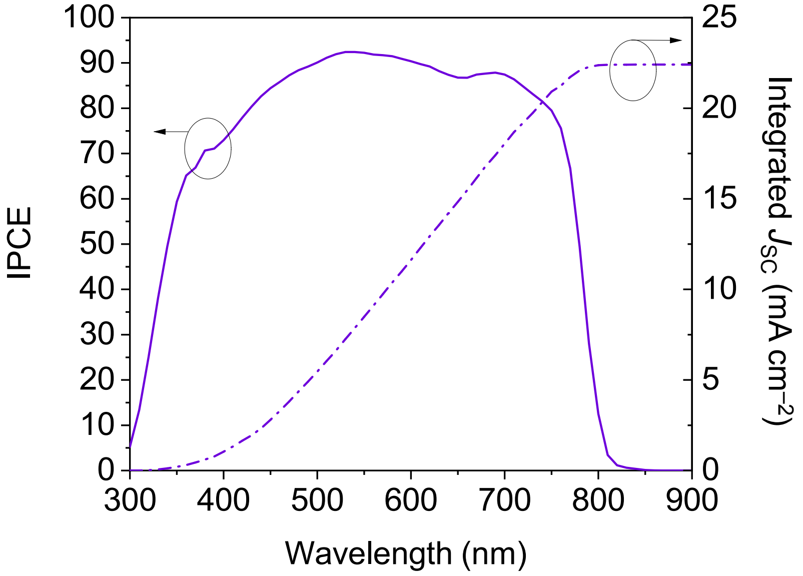

Figure S19. IPCE spectrum and integrated J_{SC} curve of n-i-p PSC fabricated with cross-linked **V1382**/dithiol (1.0 mg mL^{-1}) as HTM interlayers.

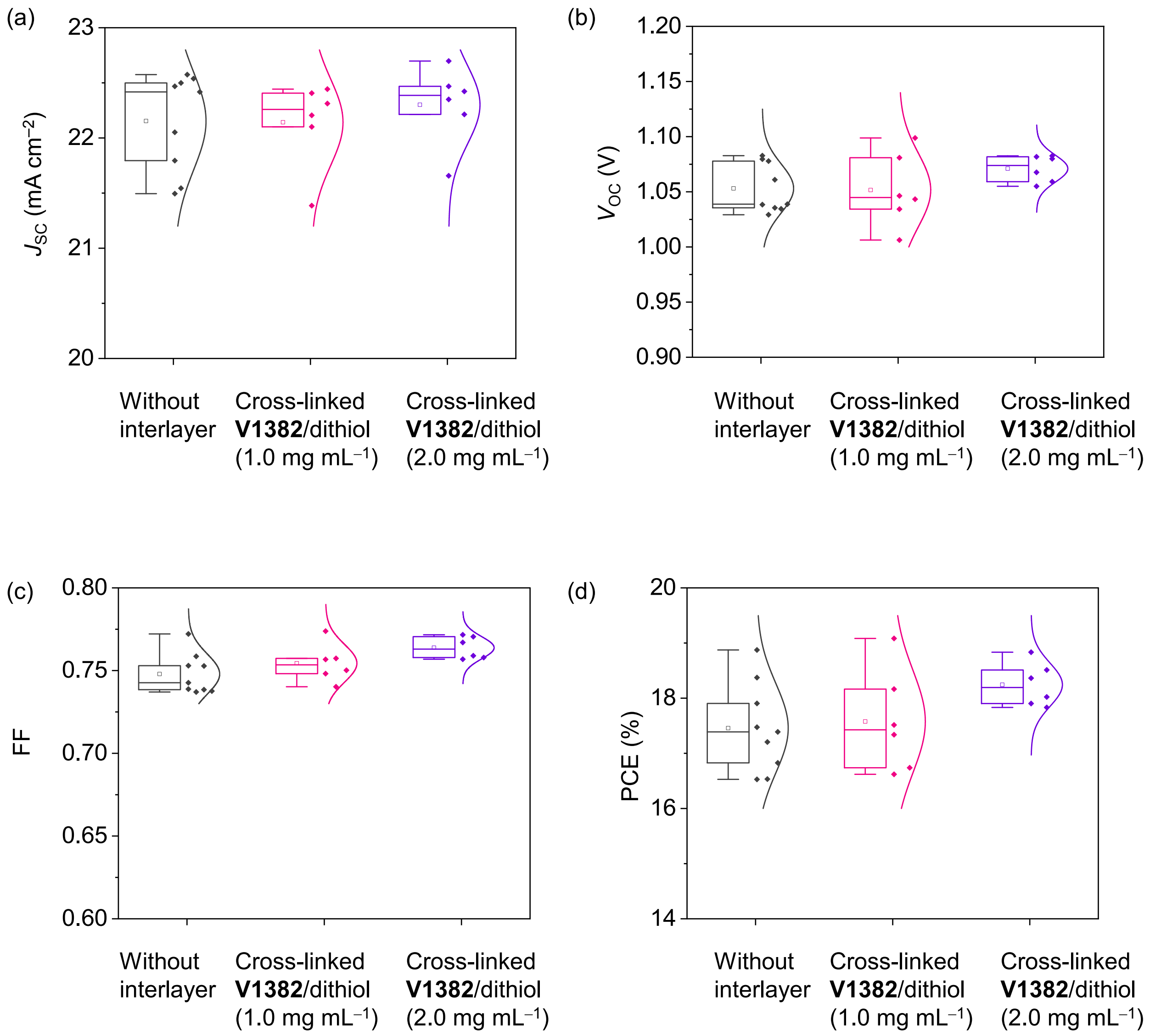

Figure S20. Box plots of (a) J_{SC}, (b) V_{OC}, (c) FF, and (d) PCE of the n-i-p PSCs fabricated without and with cross-linked **V1382**/dithiol as HTM interlayers (with different concentration of **V1382**) obtained in the forward scan.

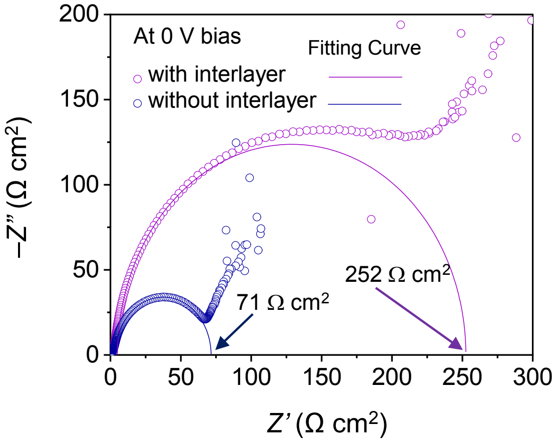

Figure S21. Complex impedance plots of n-i-p PSCs under an inert atmosphere at AM 1.5G illumination and 0 V bias.

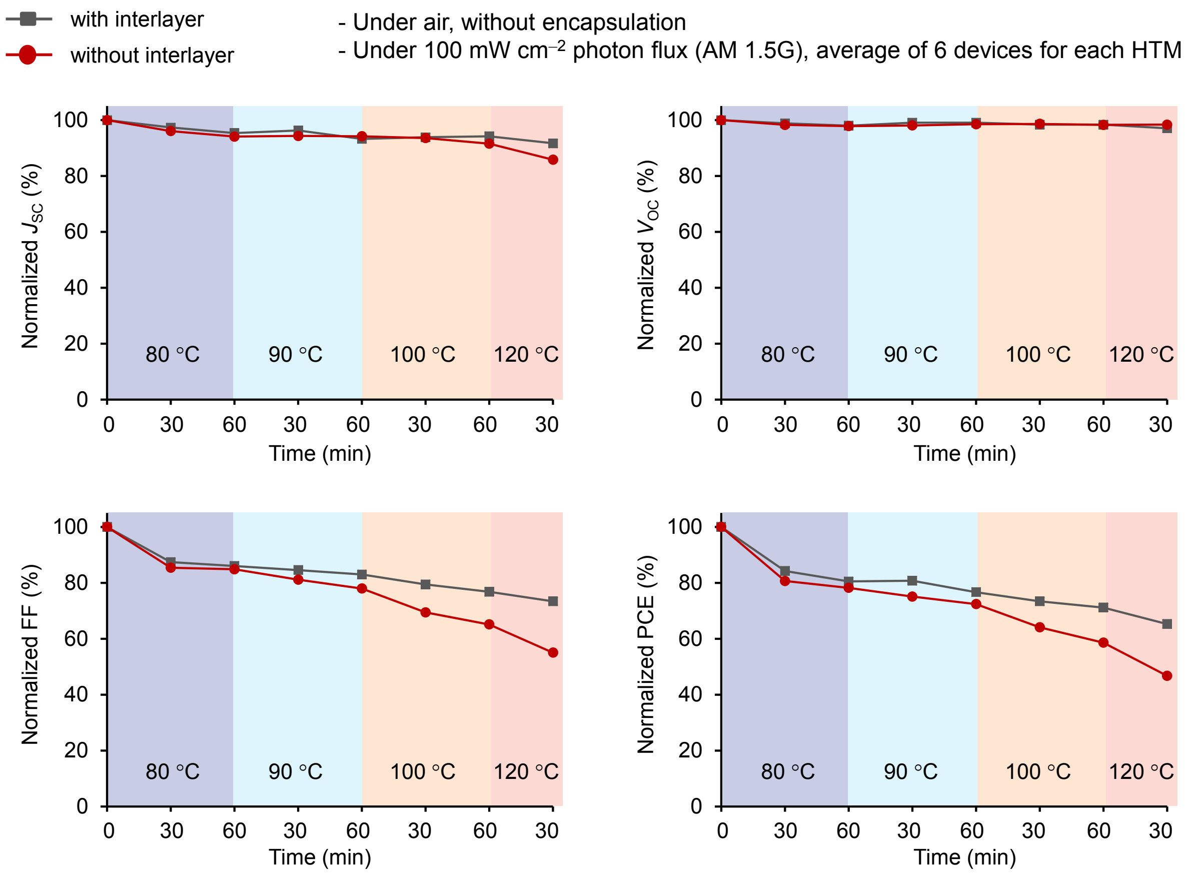

Figure S22. (a) J_{SC}, (b) V_{OC}, (c) FF, and (d) PCE evolution of the n-i-p PSCs fabricated without and with cross-linked **V1382**/dithiol as HTM interlayers under air without encapsulation, under 100 mW cm^{-2} photon flux (AM 1.5G).

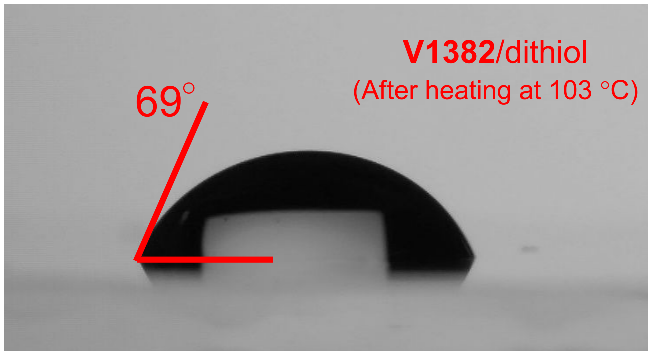

Figure S23. Contact angle of the water droplet on cross-linked **V1382**/dithiol.

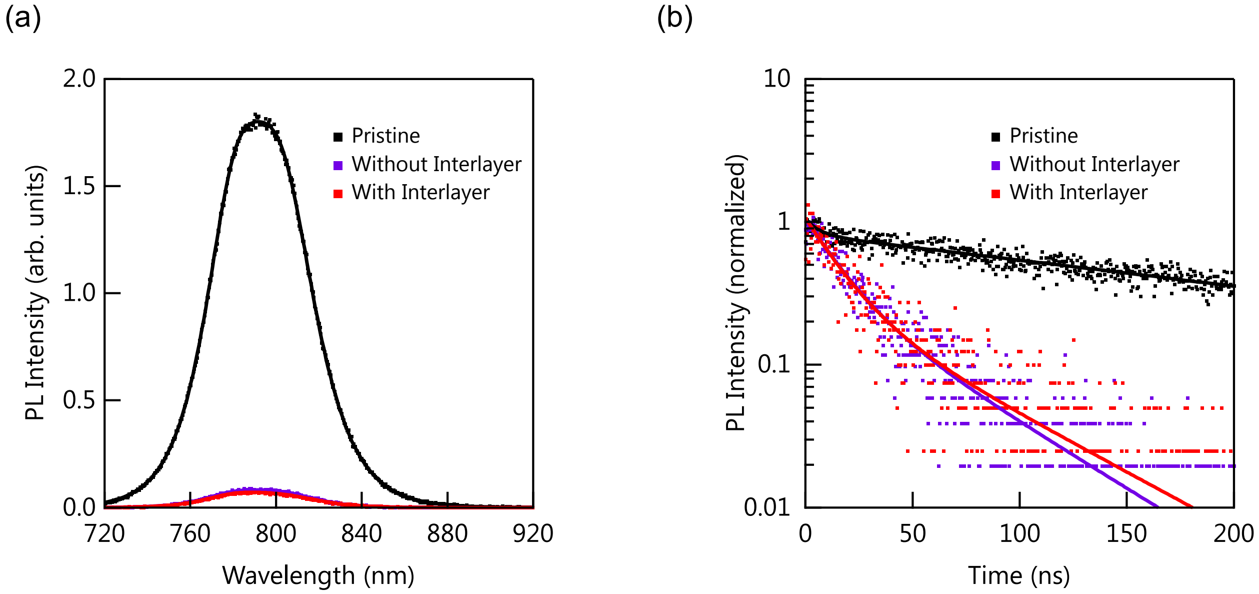

Figure S24. (a) Steady-state PL and (b) time-resolved PL spectra of the pristine perovskite ($Cs_{0.05}FA_{0.80}MA_{0.15}PbI_{2.75}Br_{0.25}$) and perovskite/without or with HTM interlayer/**Spiro-OMeTAD** films excited at 688 nm with an excitation fluence of 100 nJ cm^{-2}. The perovskite is probed through the glass side.

Table S4. PL Intensity Quenching and Fitted Decay Curve Parameters for TRPL of Perovskite Films.

Substrate	PL intensity	Life time				
		A_1	τ_1 (ns)	A_2	τ_2 (ns)	τ_{ave} (ns)
**For n-i-p**:						
Quartz	100%	26	6	113	240	196
Without interlayer	5%	35	15	16	47	25
With interlayer	4%	29	15	11	54	26
**For p-i-n**:						
Quartz	100%	26	6	113	240	196
**PTAA**	56%	30	26	38	194	120
Cross-linked **V1382**/dithiol	35%	48	14	33	164	75

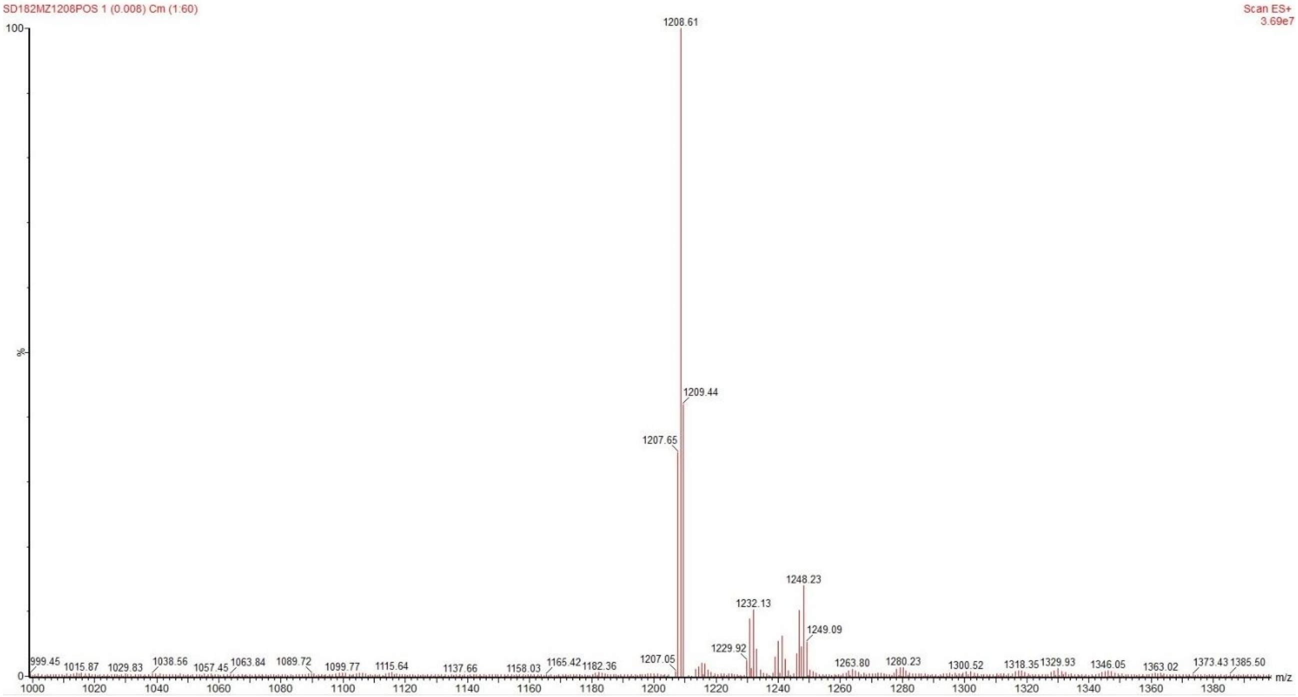

Figure S25. MS (ESI) spectra of **V1382**.

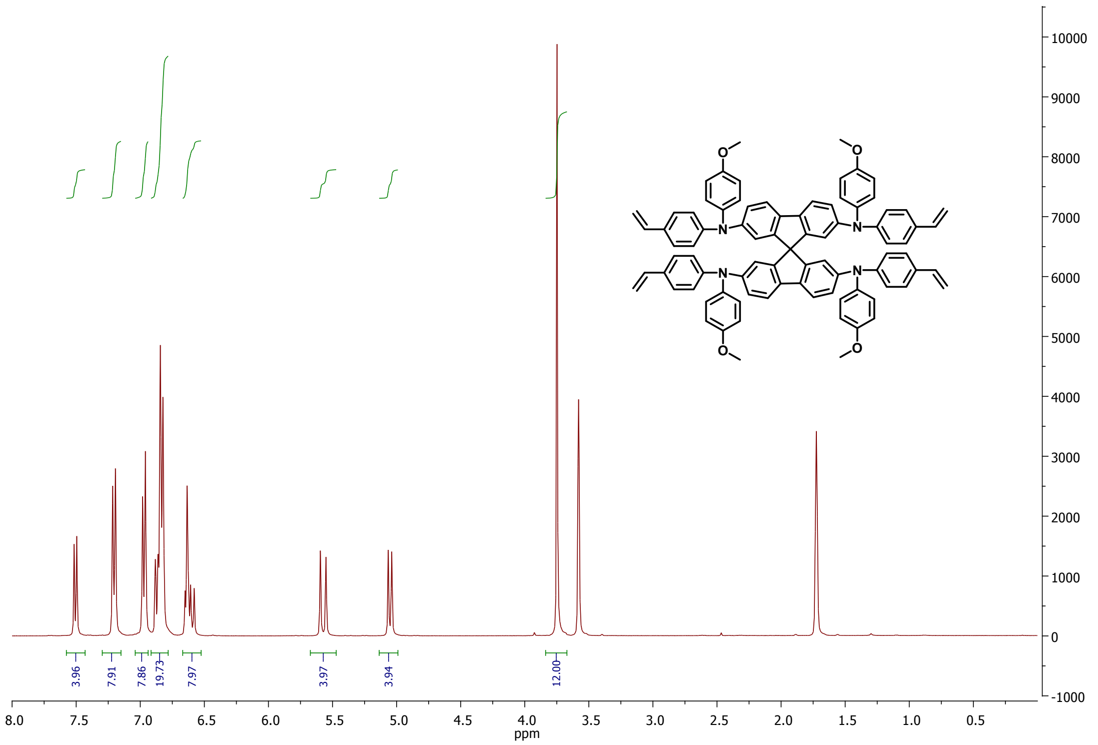

Figure S26. ^{1}H NMR (400 MHz, THF-d_6) of **V1382**.

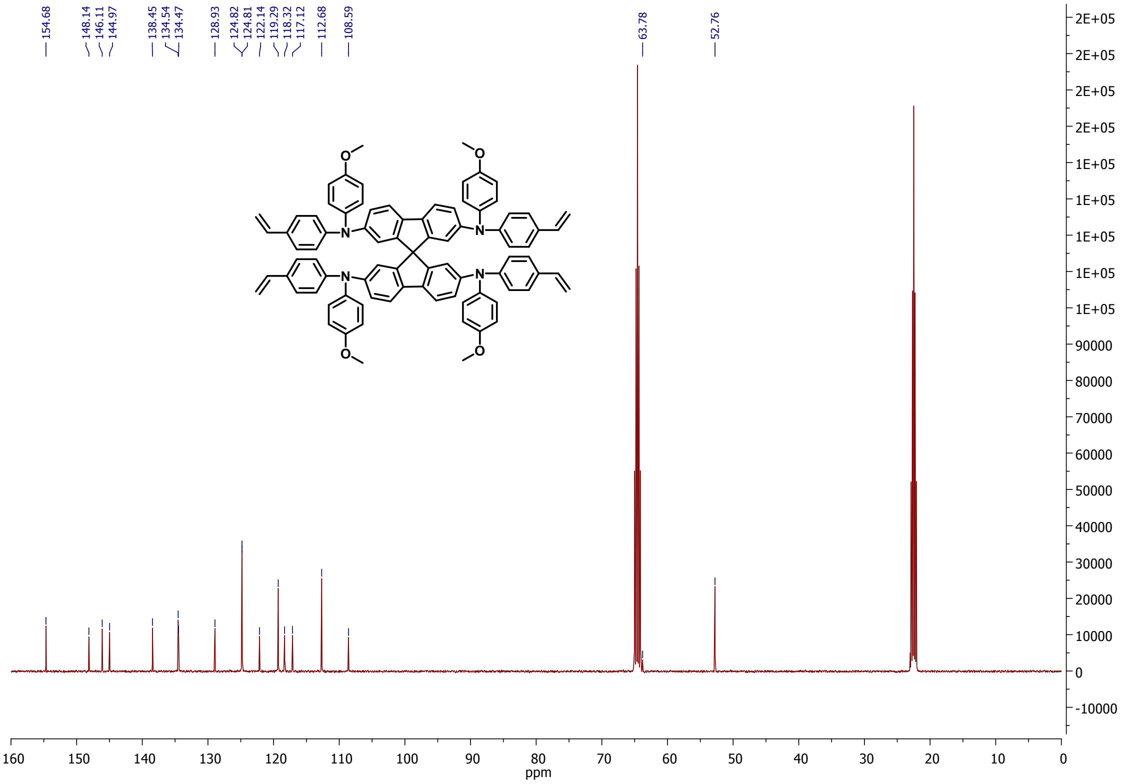

Figure S27. ^{13}C NMR (101 MHz, THF-d_6) of **V1382**.

References:

[1] M. A. Jameel.; T. C.-J. Yang.; G. J. Wilson.; R. A. Evans.; A. Gupta.; S. J. Langford. *J. Mater. Chem. A* **2021**, *9*, 27170.

[2] M. Ozaki.; Y. Ishikura.; M. A. Truong.; J. Liu.; I. Okada.; T. Tanabe.; S. Sekimoto.; T. Ohtsuki.; Y. Murata.; R. Murdey.; A. Wakamiya. *J. Mater. Chem. A* **2019**, *7*, 16947.

[3] T. Nakamura.; S. Yakumaru.; M. A. Truong.; K. Kim.; J. Liu.; S. Hu.; K. Otsuka.; R. Hashimoto.; R. Murdey.; T. Sasamori.; H. D. Kim.; H. Ohkita.; T. Handa.; Y. Kanemitsu.; A. Wakamiya. *Nat. Commun*. **2020**, *11*, 3008.

[4] M. A. Truong.; T. Funasaki.; L. Ueberricke.; W. Nojo.; R. Murdey.; T. Yamada.; S. Hu.; A. Akatsuka.; N. Sekiguchi.; S. Hira.; L. Xie.; T. Nakamura.; N. Shioya.; D. Kan.; Y. Tsuji.; S. Ikubo.; H. Yoshida.; Y. Shimakawa.; T. Hasegawa.; Y. Kanemitsu.; T. Suzuki.; A. Wakamiya. *J. Am. Chem. Soc.* **2023**,*145*, 7528.

[5] M. A. Truong.; H. Lee.; A. Shimazaki.; R. Mishima.; M. Hino.; K. Yamamoto.; K. Otsuka.; T. Handa.; Y. Kanemitsu.; R. Murdey.; A. Wakamiya. *ACS Appl. Energy Mater.* **2021**,*4*, 1484.
